# Computational flow cytometry of planktonic populations for the evaluation of microbiological-control programs in district cooling plants

**DOI:** 10.1038/s41598-020-70198-5

**Published:** 2020-08-06

**Authors:** J. M. W. R. McElhinney, A. Mawart, R. S. S. M. Alkaabi, H. S. S. Abdelsamad, A. M. Mansour, A. Hasan

**Affiliations:** 1 Applied Genomics Laboratory, Department of Biomedical Engineering, Khalifa University, Masdar Campus, Building 1A, 2st Floor, Po Box 54224, Masdar City, Abu Dhabi, UAE; 2 Center for Membranes and Advanced Water Technology (CMAT), Khalifa University, Abu Dhabi, UAE

**Keywords:** Microbiology techniques, Water microbiology

## Abstract

Biofouling poses a serious concern for the district cooling (DC) industry. Current industry practises for monitoring biofouling continue to rely on culture-based methods for microbial enumeration, which are ultimately flawed. Computational flow cytometric (cFCM) analyses, which offer enhanced reproducibility and streamlined analytics versus conventional flow cytometry were applied to samples taken from 3 sites in each of 3 plants over a 5-week sampling program. We asked whether the application of cFCM to monitoring planktonic community dynamics in DC plants could be able to provide sufficient information to enhance microbiological-control strategies at site and inform about plant performance impacts. The use of cFCM enabled the evaluation of biocide dosing, deep cleaning treatment efficiencies and routes of microbial ingress into the studied systems. Additionally, inherent risks arising from the reintroduction of microbiological communities into recently cleaned WCT basins from contaminated cooling waters were identified. However, short-term dynamics did not relate with plant performance metrics. In summary, the insights offered by this approach can inform on plant status, enable evaluations of microbial loads during biofouling mitigation programs and, ultimately, enhance industry management of the biofouling process.

## Introduction

Hot urban climates requires extensive cooling to provide habitable indoor environments and high demand in electricity and water use. Centralized production and distribution of cooling waters from district cooling plants (DCPs) is the preferred means of providing cooling waters for industrial, commercial and residential use as it is generally more economical than conventional on-site air-based cooling. The current annual energy demand for cooling accounts for 10% of global electricity use and is expected to triple by 2050, largely due to population expansions in developing hot-climate countries, including the Middle East^1^. Accordingly, the Gulf Cooperation Council (GCC) has seen a rapid expansion in district cooling facilities to meet air-conditioning requirements which are the largest such requirements in the world (in excess of 25,000,000 refrigeration tons (RT)^2^). Indeed, the peak consumption in the United Arab Emirates (UAE) is second only to Saudi Arabia in the GCC with the majority of electricity loads (~ 70%) being required for cooling^3^. Furthermore, peak cooling demands are forecast to reach 100 M RT in the GCC region by 2030^4^. Given the continued expansion of the global population, climate change and lack of alternatives for cooling in high-density urban areas, we can expect a continued reliance on DCPs for the foreseeable future. It is therefore essential that these cooling facilities are operating as efficiently and sustainably as possible in order to meet the demand for cooling, whilst mitigating environmental impacts.

District cooling systems are composed of four principle components: a make-up water (MUW) supply line, a centralized Cooling Plant, a chilled water supply network and the consumer systems. Water is the most common cooling medium for DCPs, due to its relatively low cost, high thermal storage capacity and heat transfer properties^5^. DCPs rely on evaporative cooling, consequently dissolved solids within the process water are concentrated and must be removed from the system (blowdown). Fresh make-up water (MUW) is added to replenish water lost from the system, consequently, DCPs account for a substantial proportion of industrial freshwater consumption. This is especially so in the Middle East, where the availability of freshwater is limited and demands for DCP are highest.

The performance of DCPs can be adversely impacted by a variety of issues, chief amongst these are scaling, (bio)fouling and corrosion^6^. These water-wet systems are usually maintained between 25 and 30 °C and are therefore particularly challenged by the inevitable growth of microbial communities^7^. Ingress of microbes into the system invariably leads to the colonization of surfaces by planktonic cells and subsequent development of biofilm communities^8^. The development of these biofilms (the biofouling process), are thought to hinder DCP operations by increasing the required use (and therefore costs) of treatment chemicals, reducing heat transfer efficiency of heat exchangers, reducing flow, increasing pump demands and promoting microbially-influenced corrosion (MIC)^9^. These issues can therefore be expected to raise energy and water use within the system. These issues are typically most evident in the make-up water (MUW) supply line and DCP as the chilled water supply network and consumer systems are typically in a closed loop, where microbial ingress is limited.

**Figure 1:**
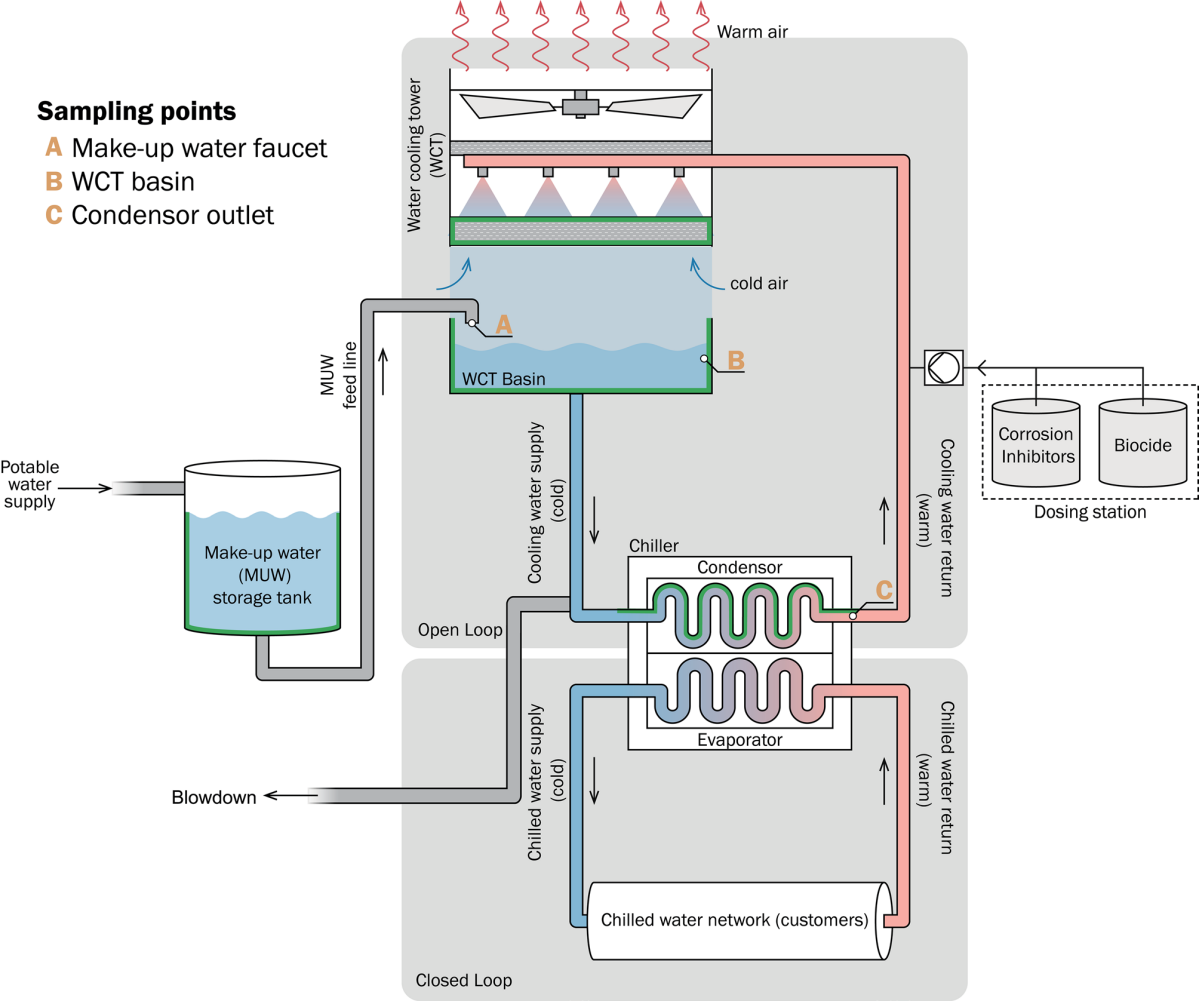
Simplified schematic of a DCP, indicating the positions of where water samples containing planktonic populations were collected for this study. Potable water feeds the make-up water (MUW) tank which, in turn, supplies the system with cooling water via the water-cooling tower (WCT) basins. Cooling water enters the chiller condenser where it is heated in condenser tubing via thermal exchange with the refrigerant that cools the chilled water returning from the customer network. Warm cooling water exits the condenser and is pumped to the WCTs where it is cooled by evaporative action. The sampled locations are: (A)—MUW faucet, (B)—Water cooling tower basins (WCT) and (C)—Condenser outlet water. Common fouling sites (MUW tank, WCT basin and condenser tubing) are highlighted in green.

As an inevitable process for open systems, biofouling requires careful and constant mitigation and management. Current industry practices for biofouling control are based on the dosing of biocides, alongside other treatment chemistries (notably dispersants, corrosion inhibitors and anti-scaling agents)^8^ and removal of biomass from the system by blowdown and deep cleaning (mechanical and targeted chemical cleaning of system parts). The application of chemistries needs to be carefully tuned to avoid excessive discharge of these often environmentally hazardous compounds whilst maximising their protective effects^8^. The monitoring of biofouling is usually based on visual inspection (common hotspots for fouling include the MUW storage tank, water cooling tower (WCT) basins and condenser tubing (Fig. 1)) and ATP or culture-based microbial enumeration. However, these monitoring approaches are hampered by the limited frequencies at which they can be carried out. These approaches have also been criticized due to the often-unclear relationship between ATP or colony forming units with the population under investigation^10^. Since culture-based methods are also hampered by the inability to cultivate ~ 99% of microbes outside of their normal environment, culture-independent methods are recommended^11^. Whilst the sessile biofilm portion of the resident communities in DCPs are responsible for biofouling issues, accurate quantification of the biofouling community is time consuming and challenging given that the majority of key surfaces in the open loop (e.g. heat exchangers) are inaccessible for sampling.

Flow cytometry (FCM) is a culture-independent method and an ideal means to quantify planktonic populations of microbes^10^. Indeed, FCM has been successfully applied to microbial monitoring programs in a variety of industrial settings, including wastewater treatment plants, drinking water plants and cooling towers^12–15^. The utility of FCM stems from its high-throughput capacity to directly measure microbial populations present in the water, and ability to provide information (e.g. for process water treatments) by discriminating cells from debris and determining their membrane integrity. One drawback of FCM is the reliance on user-guided gating (selecting cut-offs for the FCM populations of interest, based on physical or fluorometric parameters), which hinders reproducibility and increases data analysis timeframes, particularly for large datasets^16^. Research in the medical sector has led to the development of computational FCM (cFCM), wherein population statistics are obtained through computationally-determined binning or gating, thereby improving reproducibility and streamlining data analytics^17^. However, there remain few instances of cFCM in the literature and, to our knowledge, cFCM has yet to be applied to industrial process waters.

In this work, cFCM was applied to samples collected from 3 locations for each of 3 DCP systems for a 5-week sampling period with the aim of establishing whether short term planktonic dynamics could be a viable means to inform DCP management in the evaluation of their microbiological-control programs. The first part of the study focussed on describing an overview of the plants via water chemistry parameterisation and supervisory control and data acquisition (SCADA) data analysis. The corresponding planktonic populations were then enumerated within and between DCPs to establish the typical microbial loading. Temporal dynamics in these populations were then explored in relation to plant performance and microbiological-control activities to evaluate the potential of cFCM for providing insight into the microbiological loads of DCP systems for enhanced management decision making.

## Results

### Overview of plant performances

**Figure 2:**
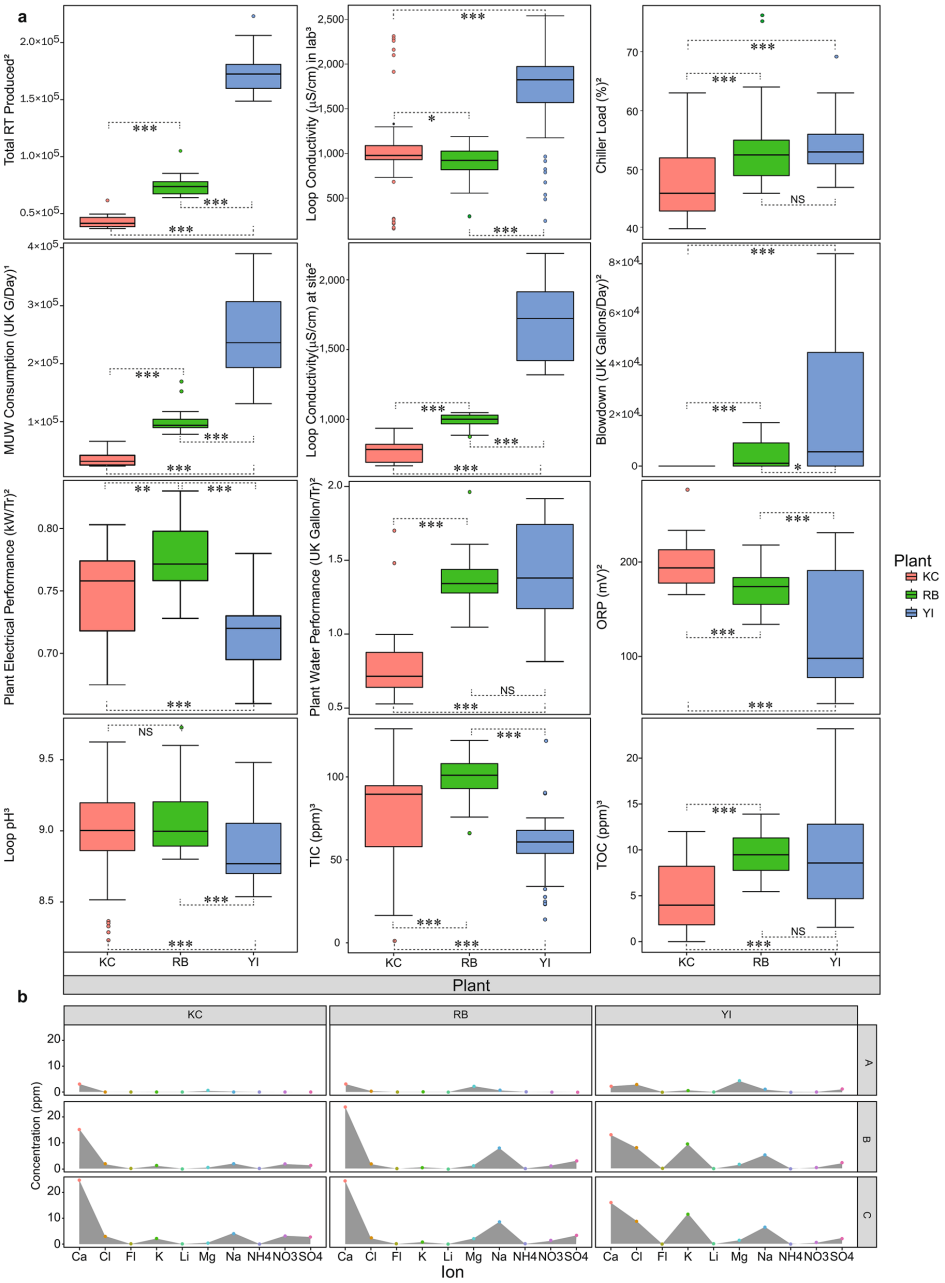
Overview of plant characteristics and water qualities highlights unique traits for the sampled DCPs. (**a**)—Boxplots comparing plant characteristics during the study period. Box boundaries represent the 25th (lower) and 75th (upper) percentiles (Q1 and Q3 respectively), upper whiskers show Q1—1.5 × interquartile range (IQR), lower whiskers show Q3 + 1.5 × IQR, data beyond those ranges are shown as individual points. Dashed lines and asterisks indicate the significance of differences in pairwise comparisons as calculated by Dunn’s test with Benjamini–Hochberg correction, where ***—*P* < 0.001, **—*P* < 0.01 *—*P* < 0.05 and NS—not significant. Depending on the parameter, certain data are available or relevant to either site B (^1^), site C (^2^) or both sites B and C (^3^) as indicated on the *y-*axis labels. (**b**)—Ion profiles in the sampled water, showing mean values for each ion (coloured by ion type). Data for each plant (KC, RB and YI) are segregated by column whereas data for sites (A, B and C) for each plant are segregated by row. The areas beneath the means have been shaded in grey to highlight ionic profiles for each site per plant for ease of visual comparison (for a more comprehensive overview of the ionic profiles please refer to Supplementary Fig. S1 and Supplementary Table 1). Data for Br, NO_2_ and PO_4_ are omitted as these could not be detected at any point across the dataset. N = 126 (36 for KC, 45 for each of RB and YI).

The DCP metadata were collected in order to establish an overview of the studied systems (Fig. 2a). In all plants, chiller loads were maintained around 48–55% during the study. YI produced the largest RT and ran the most efficiently in terms of electrical consumption per unit RT. RB, by contrast, performed slightly worse in terms of electrical performance (mean of 0.78 ± 0.03 kW/RT, with *P* < 0.001 against the means of the other plants 0.75 ± 0.04 kW/RT for KC and 0.72 ± 0.04 kW/RT for YI) but for the other metrics was typically found between KC and YI. The enhanced electrical performance at YI is thought to be the result of the application of a TES tank, unique to this plant. However, this plant used the most water per unit RT and, with a mean of 1.40 ± 0.34 UK gallons/RT (compared with 0.84 ± 0.32 at KC and 1.38 ± 0.20 at RB), was the least water efficient. This is reflected in the water consumption metrics (MUW and blowdown) which were significantly higher than the other plants (*P* < 0.05). In contrast, KC did not blowdown and used relatively low MUW during the sampling period (~ 35% of RB and ~ 14% of YI, *P* < 0.001), which enabled an examination of the effect of water use on planktonic cell populations ("Interaction between ICCs and plant operational factors" section  and Supplementary Information).

### Plant water chemistry

DCP water samples were analysed daily either onsite (ORP and loop conductivity) or, during the first and last week of sampling, in the lab (TIC, TOC and ion profiling). A comparison of water pH, conductivity and oxidation–reduction potential (ORP) yielded significant differences in contrasts between YI and the other plants (with *P* < 0.001 in all cases) suggesting that the excessive water consumption at YI, stems from low quality water automatically triggering the blow down valves (which are controlled by on-site conductivity). Conductivity on site is only measured in cooling water loop and so measures for sites A, B and C were taken in the lab (Supplementary Table 1). Conductivity values in lab and on-site datasets were generally similar but lab readings were higher for KC, indicating a possible probe issue at site. TIC loads were measured as a proxy for carbonate (a common component of mineral scales), and were highest in plant RB (mean of 72.089 ± 40.736 ppm), with *P* < 0.001 versus each of the other plants, suggesting that scaling was responsible for reducing RB plant performance. Whereas TOC remained low throughout the study (0–23.2 ppm across the plants). Chemical parameterization of the MUW entering the three plants shows that the water entering the system is consistently of high quality (Supplementary Fig. S1 and Fig. 2b). Samples collected from within the open loop of the three plants showed a consistent decrease in water quality from A (MUW) to B (WCT basins) or C (condenser outlets), with 4 ions (Ca, Cl, K and Na), conductivity, TIC and TOC increasing significantly (*P* < 0.001 in all cases) in all study plants. Of the 13 ions tested, 6 (Ca, Cl, K, Mg, Na, NO_3_ and SO_4_) comprised the bulk of ionic contaminants, with calcium being the most abundant. In agreement with the TIC levels, RB had the highest concentration of calcium, which suggests calcium carbonate, a common scaling agent^18^ is probably the main scaling agent in this system.

**Table 1:** Characteristics of the 3 DCPs sampled in this study.

Parameter	Plant		
	KC	RB	YI
Plant age at T_0_ (days)	3920	3128	3519
Plant capacity (RT)	37,500	45,000	50,000
Open loop capacity (M_3_)	1960.1	2800	ND^1^
No. of Chillers	15	18	20
No. of systems^2^	1	2	1
No. of cooling towers	10	9	8
Chiller models	York MAXE (YKYCY2J75DLES)	York MAXE (YKYCY2J75DLFS)	York MAXE (YKWPW2K75DLGS)
Biocide doses/day	3–5	5	2
Biocide type^3^	MiOx	MiOx	MiOx
Corrosion inhibitors^4^	PM3601 DC5801	PM3601 DC5801	PM3601 DC5801 CT4040
Biocide conc. (ppm)^5^	1	1	1
Biocide dosing pump flowrate (mL/min)	542.6	542.6	536.3
Serial or parallel system^6^	Parallel	Serial	Serial
TES tank	No	No	Yes

^1^ND—not determined.

^2^Number of physically separated open loop systems (c.f. Fig. 1).

^3^MiOx is the brand name of a system for on-site electrochemical generation and distribution of oxidative biocides in the open loop.

^4^Product names for the corrosion inhibitors applied at site.

^5^Intended concentration.

^6^Configuration of water flow between chillers in the open loop.

### Spatial dynamics in microbial loading of the plant systems

**Figure 3:**
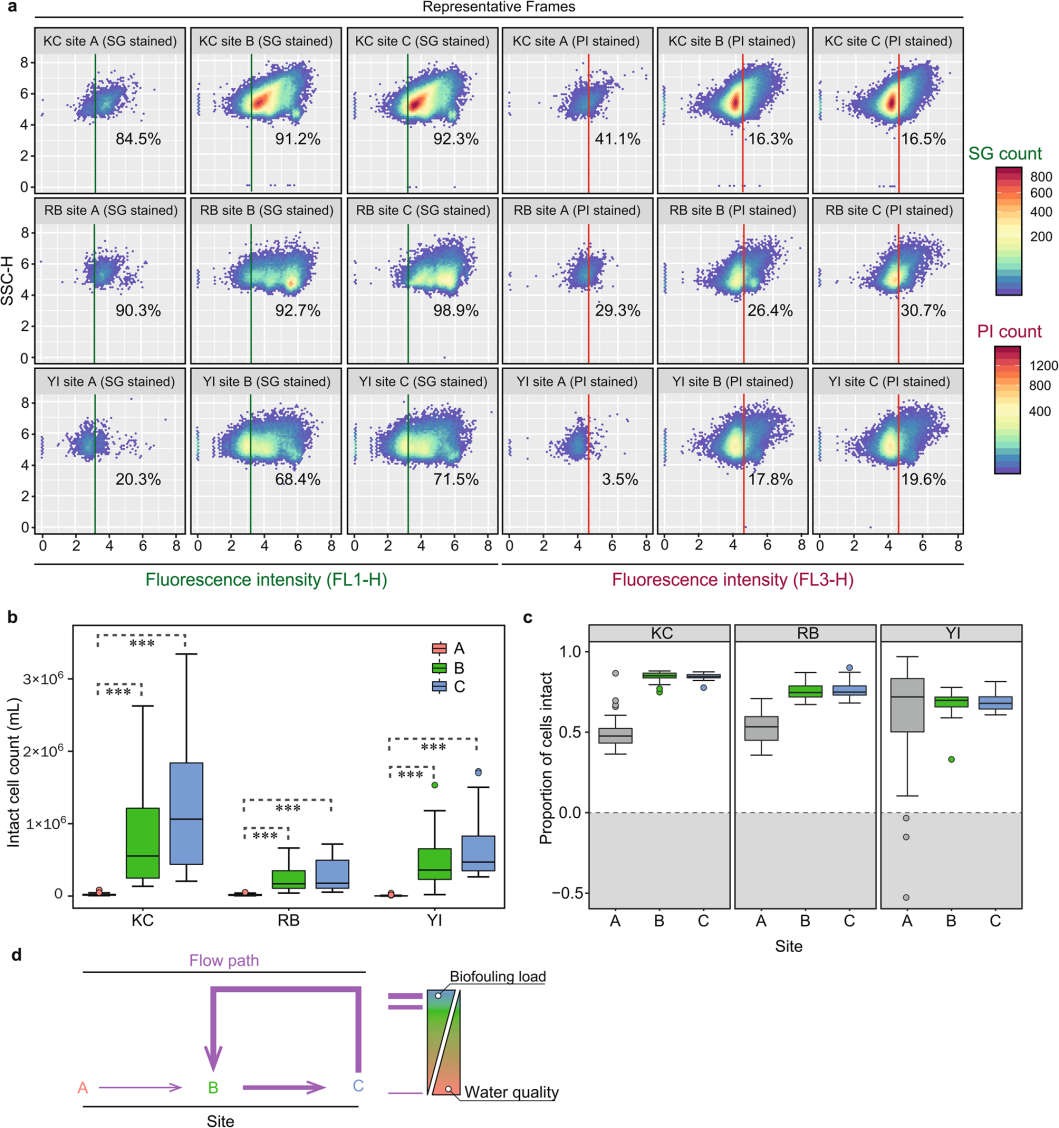
Microbes enter the system at the environment exposed WCT basins and persist in the condenser loop. **a**—representative frames from samples taken in the first day, to highlight typical gating results from each sample group across the 1244 flow frames generated in this study. **b**—Summary boxplot of intact cell counts (per mL) for all samples from each plant and site group. Box boundaries represent the 25th (lower) and 75th (upper) percentiles (Q1 and Q3 respectively), upper whiskers show Q1—1.5 × interquartile range (IQR), lower whiskers show Q3 + 1.5 × IQR, data beyond those ranges are shown as individual points. Asterisks represent significant differences between comparisons (grey dashed line) where ***—*P* < 0.001. **c**—Summary boxplot showing the proportion of living cells found at each site. Here, A site boxes are greyed out as the counts were below the limit of detection (as evidenced by the negative values obtained (grey area) when calculating these values. **d**—Conceptual model of the flow path as it relates to the microbial load and water quality highlighting results from computational gating across the cytometric dataset , i.e. that microbial ingress into the system occurs at the WCT basins persist in the condenser loop waters (site C) and are reintroduced back into the basin, where they can further contribute to fouling.

All samples were subjected to computational flow cytometric workflows to quantify microbial loads in the studied systems. cFCM enabled consistent and full population retrieval of this large dataset (1244 flow frames) in a shorter timeframe (< 1 h) than manual analysis. cFCM revealed that the majority of total event counts for open loop waters (B and C sites) across plants were positively stained by SG (82.3 ± 10.6%) and therefore identifiable as microbial cells (Fig. 3a-left panel). Whereas, PI staining (to quantify membrane-compromised populations) in corresponding samples (Fig. 3a-right panel) exhibited relatively lower percentage (19.1 ± 5.6%) of microbes with membrane-compromised cells in the open loop waters. This illustrated that the majority of the planktonic portion of cells in the sampled waters are typically found with intact cell membranes and be expected to be viable.

Sampling the three sites in each plant allowed us to examine the route of microbial ingress into the system (Fig. 3b). Samples collected from MUW faucets (A sites) showed consistently low ICCs across KC, RB and YI, with means (approaching the lower limit of detection) of 2.0 × 10^4^ (± 1.4 × 10^4^), 1.7 × 10^4^ (± 1.2 × 10^4^) and 4.8 × 10^3^ (± 7.4 × 10^3^) ICCs per mL, for KC, RB and YI respectively. The ICCs in the open CT basins (B sites) were consistently much higher than those of the MUW samples (*P* < 0.001) with means of 8.0 × 10^5^ ± 6.9 × 10^5^ (for KC), 2.4 × 10^5^ ± 1.7 × 10^5^ (for RB) and 4.6 × 10^5^ ± 3.3 × 10^5^ (for YI) ICCs per mL. Condenser outlet waters showed ICCs similar to those from the CT basins with ICC means of 1.3 × 10^6^ ± 8.9 × 10^5^ for KC, 2.9 × 10^5^ ± 2.2 × 10^5^ for RB and 6.5 × 10^6^ ± 3.9 × 10^5^ for YI. However, condenser waters showed slightly elevated counts from their WCT basin counterparts, suggesting that biofouling may be taking place in the condenser tubing. Additionally, as the basins are frequently replenished, and thereby diluted, with relatively clean MUW water (Supplementary Fig. S2), the counts in WCT samples were more variable than corresponding samples at the condenser outlets. Generally, when dilution factors are considered, the ICCs therefore correspond well with water chemistry data (Fig. 2, Supplementary Table 1 and Supplementary Fig. 2), demonstrating that water quality is a good indicator of cell loading. ICCs also corresponded well to the absence of biofilms in the MUW tanks and visible extent of biofouling in the WCT basins of the studied systems.

The ratio of living cells was 0.66 ± 0.19 at YI, 0.68 ± 0.13 at RB and at KC was slightly elevated at 0.73 ± 0.18 (*P* < 0.001). Living cell ratios remained consistent between WCT basin and corresponding condenser outlet samples (Fig. 3c), suggesting that microbial populations retain viability when entering the condenser loop. This microbial loading can be expected to adversely affect performance by forming biofilms (and subsequent MIC) on functional condenser tubing (thereby reducing heat transfer efficiency and chiller lifespan). Despite the high retention times in MUW storage tanks (~ 1–2 weeks, based on daily MUW usage), microbial ingress into the DCP seems to occur primarily at the open WCT basins. Since the MUW had a consistently low density of cells, MUW samples were not investigated further. Given the flow path of these DCP systems, the correspondence between ICCs in WCT basins and condenser outlet waters is perhaps unsurprising. However, the recirculation of water between these locations may exacerbate biofouling due to the reintroduction of viable cells from within the open loop back into the basin (Fig. 3d). Of the 3 study plants, KC exhibited the highest overall counts throughout the study period (7.0 × 10^5^ ± 8.3 × 10^5^, where *P* < 0.01 versus YI and *P* < 0.001 versus RB), followed by YI (3.7 × 10^5^ ± 4.0 × 10^5^, where *P* < 0.05 versus RB) and with RB exhibiting the lowest counts (1.8 × 10^5^ ± 2.0 × 10^5^) over the period (Fig. 3b). These counts were next examined against operational features to establish whether they were determined by plant management practises or stochastic ("Interaction between ICCs and plant operational factors" section).

### Interaction between ICCs and plant operational factors

DC plants are complex systems that are influenced by, and regulated based on, a variety of factors. We therefore sought to establish the key parameters driving observations of microbial loads and, in turn, how these observations could assist with biofouling mitigation efforts.

#### cFCM can guide oxidative biocide dosing programs

**Figure 4:**
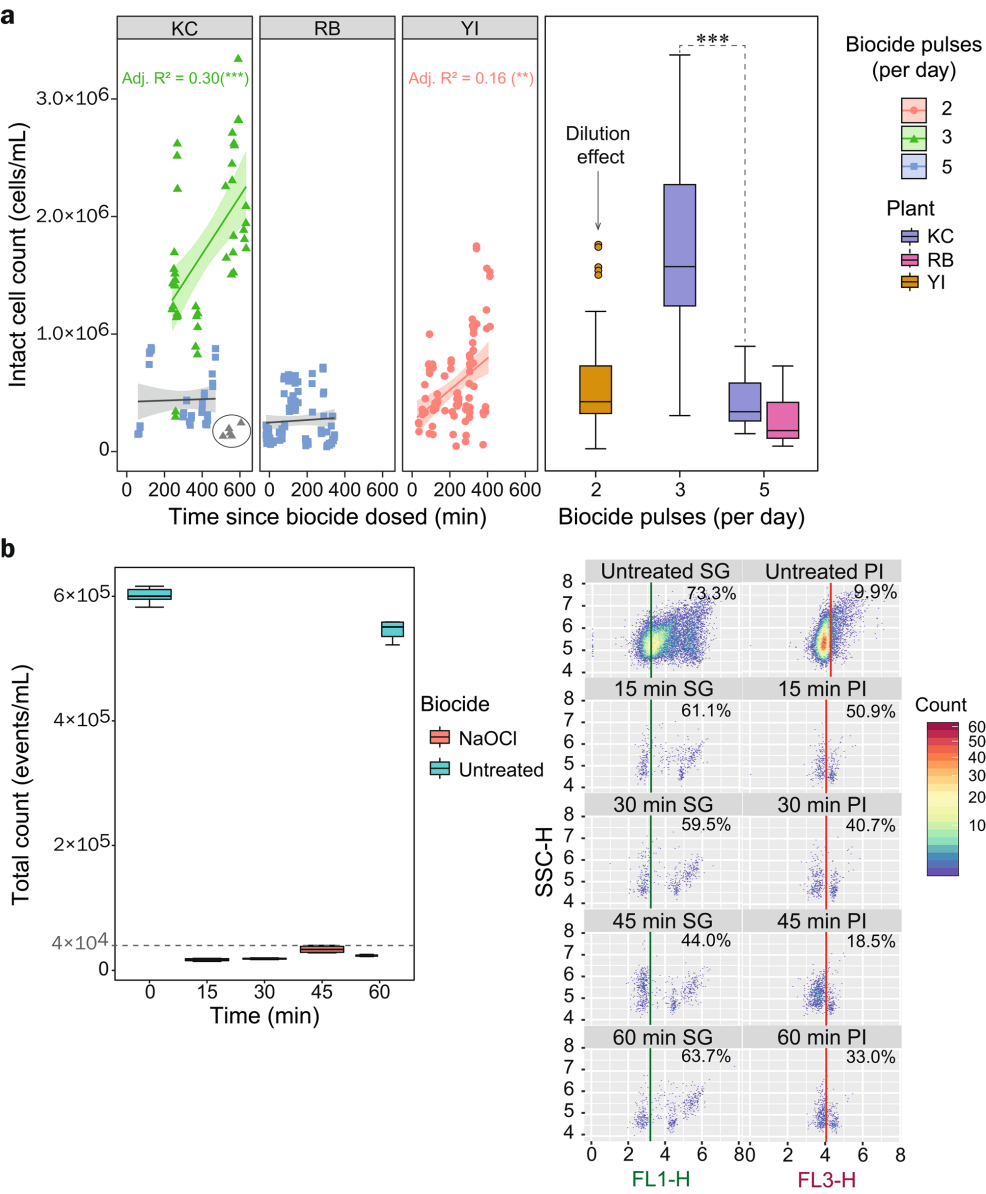
Recovery of microbial populations can be mitigated by additional pulses of biocide treatments. **a** Right panel—Intact cell counts across the three study plants, as a function of time since biocide dosed, showing linear regression fits (stratified by the frequency of daily biocide treatments (pulses) per day, illustrating cell recovery (and a reduced recovery rate when biocide is applied more frequently (5 pulses per day). Grey points in KC were omitted from the regression as they were collected in basins prior to reconnection (see section entitled “Deep cleaning operations of WCT basins can be negated by the reintroduction of microbes from the open loop” for further information). Left panel—box plot representation of the right panel, showing the distribution of counts in relation to biocide pulse frequency. The reduced count in YI (2 pulses per day) is thought to be a consequence of higher MUW usage at this plant. Asterisks indicate significant differences between groups (where ***P < 0.001) as determined by Kruskal–Wallis with Dunn’s post hoc test (and Benjamini–Hochberg correction). **b**—Time course assay of 1 ppm NaClO biocide treatment of KC WCT samples and corresponding dot plots (SSC-H vs FL1-H or FL3-H) of sample events throughout the time course.

**Figure 5:**
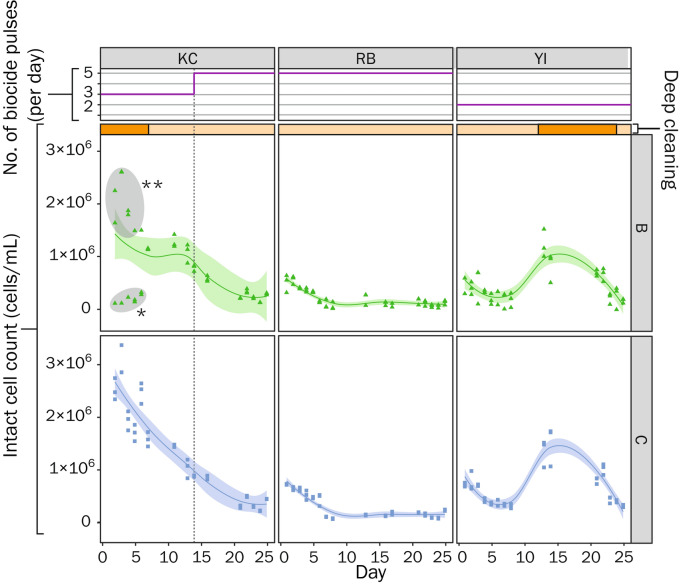
Spatiotemporal dynamics in living cell counts of the planktonic microbial communities in DCPs over the sampling period as they relate to biocide treatments and deep cleaning events. Cytometric counts of living cells (SG + ve counts × proportion of PI +ve events) are shown for sampling points B—cooling tower (CT) basins and C—Condenser outlets across all study plants (KC, RB and YI). Data have been fitted with Loess curves to highlight trends in microbial abundance with time, where the bands show the predicted 95% confidence interval. The incidence of deep cleaning events (wherein biofilms are mechanically removed from the cooling basins) are shown as orange blocks within the yellow bars (deep cleaning at RB was completed approximately 2 weeks prior to the first day of sampling). The line charts below show the number of biocide pulses (40 min treatments with oxidative biocide mixtures) per day. The dotted line through plots of living cells in KC (sites B and C) indicate the sampling day where biocide pulses where increased from 3 to 5 daily treatments. *KC WCT basin samples that were taken from basins that had undergone deep cleaning but not yet been reconnected to the system. **KC WCT basin samples that were taken from WCT blocks that had yet to undergo deep cleaning (> 200 days since last clean).

Each plant uses a mixed oxidant biocide treatment which is dosed in 40-min pulses between 2–5 times per day. When evaluating ICCs in relation to the time since biocide was last dosed into the system (Fig. 4a), it can be seen that the number of cells in DC biofouling communities quickly (< 10 h) begin to recover when pulse frequency is < 5 times per day. In contrast, cell recovery was not observed when biocide pulse frequency was set to 5 pulses/day, demonstrating a sustained control of planktonic populations through a higher residual of biocide. The change in biocide pulse frequency at KC enabled us to evaluate the impact of an increased pulse frequency in a single system (boxplot in Figs. 4a and Fig. 5). This change in dosing resulted in a significant drop in microbial loads, from a mean of 1.5 × 10^6^ ± 8 × 10^5^ cells/mL in the open loop, down to 4.4 × 10^5^ ± 2.4 × 10^5^ (*P* < 0.001). After 5 days from this change in biocide treatment regime (Fig. 5), ICCs at KC were in line with that of RB (which also doses 5 pulse per day). This decrease in ICC is commensurate in both WCT basins and condenser outlet waters. RB uniquely showed no visible biofilm formation in the study period and, in the days following the switch to 5 pulses per day at KC, no biofilm growth was observed. Indeed, there was clear correspondence between the ICCs at all plants with the visible levels of biofouling. However, with a residual ICC in the order of 10^5^ cells/mL, there remained substantial microbial loads in the open loops. Since resistance to chlorine-based biocides has been seen in water communities^19^, we tested the DCP biocide NaClO at the recommended concentration (1 ppm FAC) on the target planktonic population (using KC WCT basin samples) to evaluate its efficiency. Controlled dosing of NaClO resulted in an immediate (< 15 min) and steep reduction (~ 96%) in observed event counts (< 4 × 10^4^ cells/mL) in KC WCT basin samples. This finding is similar to prior FCM findings from NaClO dosing on cooling tower populations^13^ and suggests that the planktonic populations in these DCPs are not resistant to treatment. This raised the question as to whether the applied dose of biocide at site was in fact reaching the target concentration of 1 ppm FAC. The pumps at KC and RB have a flowrate of 542.6 mL/min and dose for 40 min. Therefore, the volume biocide dosed per pulse is 21.7 L, this is diluted in a loop volume of 1960.1 m^3^ (at KC) and 2800 m^3^ (at RB), the loop volume at YI could not be determined. The MiOx produces oxidative biocide at a concentration of 4500 ppm ± 1000 FAC. Consequently, the final biocide concentration is 0.05 ppm FAC (at KC) and 0.03 ppm FAC (at RB). Taken alongside the controlled dosing (Fig. 4b), this implies that there is scope to enhance the biocide dosing regimen at these DCPs by raising the concentration toward the recommended 0.5–1 ppm. This case illustrates the utility of cFCM to diagnose and evaluate water treatment dosing issues in industrial installations.

#### Deep cleaning operations of WCT basins can be negated by the reintroduction of microbes from the open loop

Each plant carries out a bi-annual deep cleaning (wherein biofilms are physically removed from the WCT basins and a chemical treatment is applied to the surface). RB completed a deep cleaning operation ~ 14 days before the sampling period, whereas deep cleaning was ongoing on day 1 of the sampling program at KC and YI started deep cleaning at the end of the sampling period. Prior to the change in biocide pulse frequency at KC (day 14), deep cleaning of WCT basins resulted in clear decreases in the count of living cells in these basins (Fig. 5), which is quickly reflected in the condenser outlet waters. In the case of KC, we can observe a high ICC in a subset of WCT basin samples at the beginning of the sample collection period. This can be attributed to these samples being collected from basin blocks that were active but had not yet undergone deep cleaning (> 200 days since last clean). This value also reflects the poorer quality of water being introduced into the open loop during the deep cleaning of other WTC basins and explains the correspondingly higher counts in KC condenser outlet samples collected during that period. For YI, the deep cleaning resulted in a marked reduction in ICCs for the limited window of time that the site was monitored post clean.

**Figure 6:**
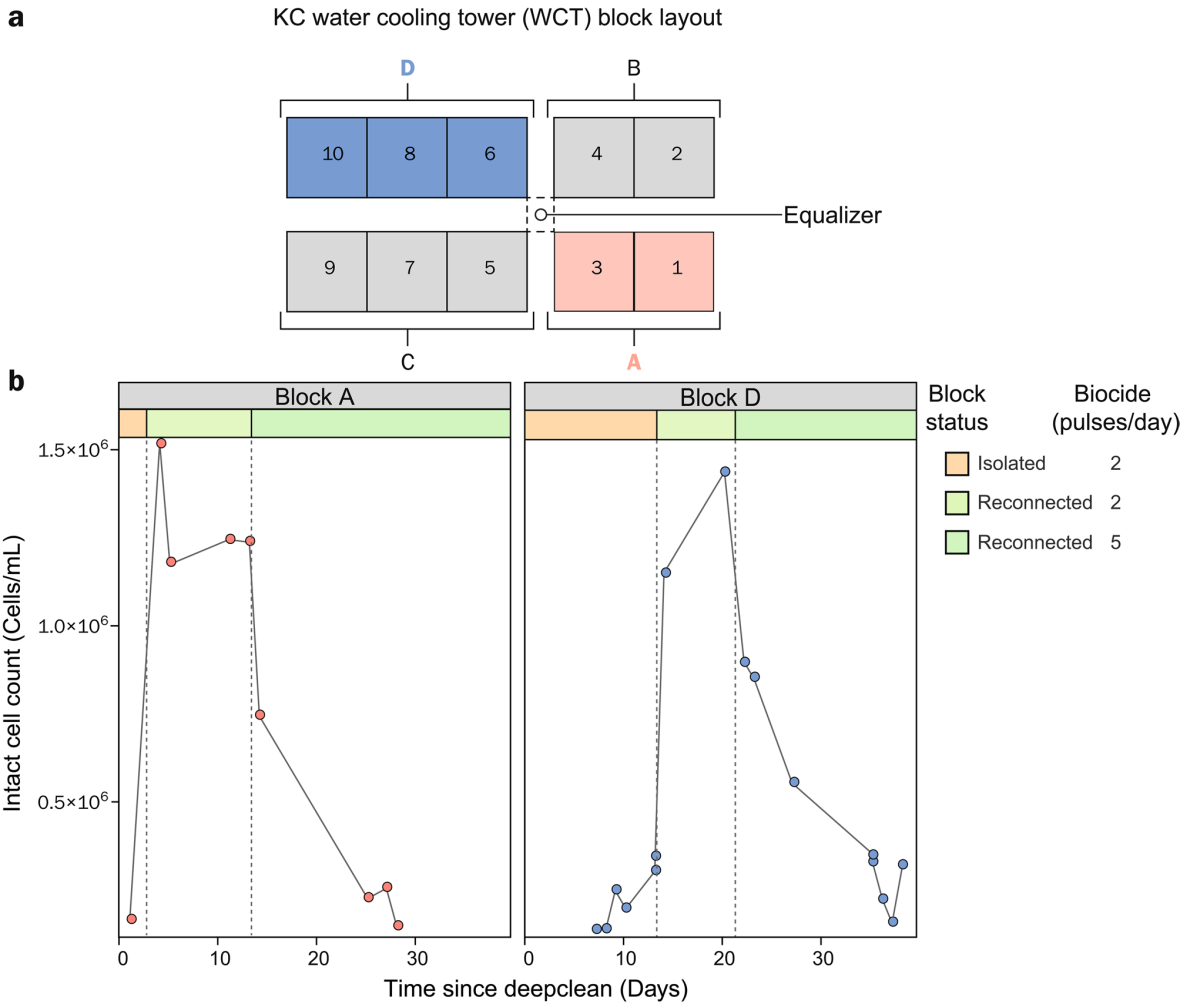
Reconnection of water-cooling towers (WCT) following deep clean treatments (physical biofilm removal) leads to rapid reestablishment of planktonic populations. **a**—Schematic showing bird’s eye view of WCT basin blocks A–D at plant KC, of which A (red) and D (blue) were sampled during the deep cleaning process. **b**—Intact cell counts from samples collected in blocks A and D before (yellow region of the plot) and after reconnection (light green) are shown in relation to time since deep clean. The reduction in counts follows the increases in biocide pulse frequency (from 3 to 5 pulses per day), as highlighted by the dark green shaded region of the plot.

KC is the only plant that underwent deep cleaning and where basins remained isolated from the system for a sufficient length of time to permit an investigation of the regrowth of microbes in recently cleaned WCT basins (Fig. 6). At KC, basins are organised into four blocks connected by a set of equalizers for balancing basin water volumes (Fig. 6a) two of which (blocks A and D), were able to be sampled during their isolation from the rest of the system as well as after activation of the associated cooling tower. At the time blocks A and D were reconnected to the system after deep cleaning, the equalizer between A-B and B-D was open and A-C and C-D were closed as C, the last block for deep cleaning was drained. Block B was the first to undergo deep cleaning few days before A and D. Block C was the last to undergo deep cleaning and was immediately reconnected, preventing sample collection before cooling tower activation or equalizer opening. We therefore focussed on Blocks A and D, in order to better understand the effect of deep cleaning on the microbial load in WCT basins. The ICCs observed immediately following the deep clean highlight the efficiency of the deep cleaning with a significant decrease in WCT basin ICCs that remained low whilst the basin was isolated from the system. However, as the condenser loop was not specifically treated during this time, a sharp increase in WCT basin ICCs was observed immediately after their reconnection to the rest of the system (Fig. 6b). This increase resulted in similar microbial loads in WCT basin to those of the corresponding condenser outlet waters at the time of reconnection. This suggests a “seeding” of the clean basin with the micro-organisms circulating in the active loop during the deep cleaning. Consequently, the long-term benefits of the deep clean are likely to be hampered by this reintroduction of microbes following reconnection. From this, it is fairly clear that the DCPs would benefit from shock dosing of condenser waters with biocide during the deep cleaning operation to mitigate this reseeding. By highlighting the limitation of deep cleaning to the basin waters, cFCM-based short-term microbial dynamics is further demonstrated to provide valuable insight that can guide biofouling management practices for industrial water process installations.

One limitation in FCM-based monitoring is that there are currently no industry benchmarks or standard methods for microbiological risk assessment based on ICCs^10^. Whilst there are guidelines for culture-dependent colony forming units (CFUs) at site (< 1 × 10^4^ CFU/mL) these values (collected as part of the routine microbial monitoring at DCPs) during the sampling period were 0.1—1 × 10^3^ CFU/mL for KC, 1 × 10^2^ CFU/mL for RB and 0.1—1 × 10^2^ CFU/mL for YI. The CFU-derived counts therefore represent only 0.01–0.06% of the corresponding ICCs observed during the study period. Moreover, the limited frequency (once per 4 weeks) and numerical resolution of CFU-based monitoring at site failed to detect the dynamics in microbial load that we observed following biocide or deep cleaning treatments. These examples add to increasing body of prior work ^13,20,21^ in further demonstrating the shortfalls in culture-based methods for monitoring biofouling and relative merits of culture-independent FCM methodologies. The application of FCM and analysis of the resulting data is becoming progressively more user-friendly and powerful, especially with the advent of cFCM. We therefore expect these tools to become increasingly applied within industrial systems in the near future. If so, it will be increasingly important that proper guidelines are established in order to facilitate informed and appropriate decision-making for microbiological-control programs in industry.

## Discussion

In this work, we asked whether cFCM analyses of process water from DCPs could inform in the assessment of water quality and microbiological-control management decisions. Our findings demonstrate, seemingly for the first time, that cFCM can be readily applied for batch analysis of large (1244 samples) cytometric datasets from environmental samples (to our knowledge, cFCM has previously been limited to relatively clean samples from medical research^17^) and can rapidly provide information-rich results for industrial microbiological-control programs.

Previous work applying FCM for biocide treatment in industrial settings has well demonstrated the advantages of FCM^13,22,23^. Such work has shown the efficacy of biocides on planktonic populations. Here we have built on prior work by demonstrating that FCM can also be useful for identifying cases where biocide dosing can be further optimised. In concordance with recently published work^24^, we also found that standard oxidative biocide dosing does not completely inactivate cooling tower communities (even when dosed at the highest permissible concentrations). It must be noted that it is well established that biofilms confer to their resident communities a degree of protection against biocides, relative to their planktonic counterparts^25–27^. Therefore, controlled biofilm sampling is advisable for DCP operators. However, if ICC data demonstrates limited efficacy of biocide treatments in the planktonic phase, then DCP system biofouling can be expected to worsen over time.

Current methodologies for direct biofouling quantitation typically rely on significant processing or lab cultivation of biofilm samples^28,29^ that may confound interpretation (although in certain cases, side line-installed corrosion coupons and bioprobes can provide insight). For biofouling to be properly quantified it is essential that in situ growth is measured over a fixed surface area in a given unit of time (such as by deployment of a biofilm sampling device, housing coupons with suitable surface characteristics) which can be impractical for DCP operators. Due to the periods required to allow sufficient growth, limited accessibility to functional surfaces and heterogeneous distribution of biomass across those surfaces, the direct quantitation of biofouling communities within these industrial systems remains an ongoing challenge.

In contrast, monitoring the planktonic population is relatively easy and carries the advantage that microbial loads can be tracked, and intervened on, before significant biofilm develops. Indeed, using cFCM-based microbial enumeration, it was possible to detect substantial microbial populations entering and exiting condenser tubing without requiring disruptive and expensive shut downs. It was also possible to evaluate community recoveries after biocide dosing. Whilst biofilms are typically more recalcitrant to biocide dosing, we observed limited efficacy in biocide dosing and were able to identify shortfalls in biocide dosing from the cytometric data. Additionally, planktonic monitoring identified biofouling risks of reintroducing system waters with high microbial loads following reconnection of WCT basins to the system after deep cleaning. This suggested the need for shock treatments in the open loop during WCT deep clean operations, further illustrating the utility of FCM in assisting with microbiological management decisions of DCP operators. However, when using planktonic measures, it is essential to be familiar with the qualities and volumes of the different water bodies in use. For instance, MUW in our study systems is high purity potable water that is introduced to the systems on an as-needed basis and, as it is cleaner than open loop waters, MUW can dilute down microbial estimates and so must accounted for when evaluating biofouling risks. Consequently, we found WCT basins to be highly variable in counts (relative to MUW and condenser outlet waters) and would therefore suggest microbial loads are monitored from a representative points within the open loop for future monitoring programs (e.g. from a header in the cooling water return line). Monitoring points immediately downstream of condenser tubing could also be established to facilitate biofouling management of individual chillers.

To our knowledge, there are, no reported attempts to quantitatively relate microbial loading to performance metrics (such as water and energy efficiencies) in DCPs. Here we attempted to do so (supplementary results), but found little predictive power in planktonic populations over the study period. Importantly, there was also little correspondence with well-established abiotic factors and plant performance during this period. Whilst longer study times may permit such assessments, these systems are influenced by a variety of interacting operational and environmental factors and so quantitation of the relative impacts of common issues (such as scaling, corrosion and biofouling) on plant performance is not a trivial issue.

Recently developed online flow cytometry, for real-time system monitoring, appears to be an especially promising tool for industry operators^12,30^. Though industry standards for acceptable microbial loads are needed for routine on site use of cFCM for biofouling management, cFCM is well suited for the monitoring of planktonic biofouling populations in recirculating cooling systems.

In summary, cFCM is a valuable tool for supporting microbiological-control programs that can guide biofouling mitigation practices for industrial water process installations.

## Materials and methods

### Plant descriptions and sample collection

Water samples were collected from three active full-scale district cooling plants (hereafter designated KC, RB and YI) daily over a 5-week sampling period. The main characteristics of the plants are described in Table 1. These plants are between 9 and 11 years old, are supplied with potable MUW and of similar scale. All 3 plants are equipped with fixed frequency centrifugal chillers from the same manufacturer but with slight differences in RT capacity. Cooling tower basins at the study sites are grouped into blocks of 2–4 basins that are connected by equalizers, which permit mixed flow within and between basin blocks. Individual basin volumes are similar in size and have maximum capacities of approximately 205 m^3^. Uniquely, RB chillers and WCT basins are segregated into two independent systems while YI includes a thermal energy storage (TES) tank. The conductivity, ORP and pH of loop waters is monitored via in situ electrochemical probes (with the exception of KC which does not follow pH). Each plant uses a MiOx dosing system which administers the same mixed oxidant biocide (composed of sodium hypochlorite and peroxide) in 40 min pulses from 2 (YI), 3 (KC) or 5 (RB) times at fixed times (between 04:00 and 12:30) per day with a final concentration target of 1 ppm free available chlorine (FAC) in the open loop. At KC, the biocide pulse frequency was increased from 3 to 5 pulses/day during the sampling program, allowing us to examine the influence of this change of the biofouling mitigation program on counts in the planktonic phase. Remaining treatment chemicals (corrosion inhibitors and anti-scaling agents) are also uniform across the study plants, with the exception that YI applies a regular dose of a second corrosion inhibitor on a weekly basis. All three plants apply a bi-annual deep clean wherein the biofilms are mechanically and chemically removed from the CT basins. The sampling program coincided with this activity for two of the study plants (KC and YI). Over the last 2 years, biofouling was observed at all three plants. However, during the study period, extensive biofilms were clearly visible in the water cooling towers and basins of KC and YI whereas no biofilms were observed in RB (which was recently deep cleaned and used a higher frequency of biocide pulses per day). Plant performance and operational metadata was collected from the supervisory control and data acquisition (SCADA) systems on site and was uploaded to Mendeley data (please see data availability statement).

### Site sampling

From each plant, 3 biological replicates were collected from each of three sampling sites per plant: (A) the make-up water (MUW) faucet, (B) cooling tower basins and (C) the condenser outlet from an active chiller (Fig. 1). Where chiller arrangements were in serial configuration (i.e. path of water was cooling tower → chiller 1 → chiller 2 → cooling tower) water was always collected from the upstream chiller of the pair. 50 mL was collected at each sampling point per samplea minimum of 5 L was drained from condenser outlet valves before sampling to avoid artefacts from stationary dead leg volumes. Basin samples were each collected from unique basins to capture a more complete picture of the planktonic community at the time of sampling. Following collection, samples were transported to the laboratory within 30 min and immediately processed for flow cytometric profiling. Samples were stored at 4 °C until water chemistry parameterization.

### Water parameterization

Conductivity and pH measures were taken on an Orion VersaStar Pro electrochemical multi parameter meter (ThermoFisher, US) on the same day of sampling. The total organic (TOC) and total inorganic (TIC) carbon was measured for site samples (8 mL vol) using an Innovox TOC analyzer (GE Analytical Instruments, USA). 30% w:v Na_2_SO_4_ was used as the oxidizer and 6 N H_3_PO_4_ as the reducing agent. TIC and TOC values were ascertained by fitting to a five-point calibration curve (1–1000 ppm) of potassium hydrogen phthalate (TIC) or sucrose (TOC) standards (GE, USA). 5 mL of site samples were filtered through a 0.22 µm surfactant-free cellulose acetate membrane and diluted to 1:5 and 1:50 v:v in type 1 water. 2 mL of diluted samples was then subjected to ion profiling. Ion profiling was carried out for 6 cations (NH_4_^+^, Ca^2+^, Na^+^, K^+^, Mg^2+^ and Li^+^) and 7 anions (Cl^-^, SO_4_^2-^, NO_2_^-^, NO_3_^-^, PO_4_^3-^, Br^-^ and F^-^) on a Dionex ICS 5000 high pressure ion chromatography (HPIC) system (ThermoFisher, USA) fitted with a trap column and IonPac CS16 (cations) and Dionex IonPac AS17-C (anions) analytical columns. HPIC was carried out at a flow rate of 1 mL/min with a column temperature of 35 °C, suppressor set to 99 mA (cations) and 117 mA (anions) and the eluent generator set to 10µS/cm for EG1 and 40 µS/cm for EG2. Samples were run with calibration verification standards before and after sample blocks and blanks (type 1 water) were placed periodically to monitor instrument performance and retention time drifts. Concentrations of ions were determined based on fitting to a 6 point (cation) and 8 point (anion) calibration curves based on 1:5 (anion only), 1:10 (anion only), 1:20, 1:40, 1:80, 1:160, 1:320 and 1:640 v:v dilutions of Dionex six cation-II or Dionex seven anion-II standards in type 1 water. Ion chromatograms were analysed in Chromeleon Software (ThermoFisher, USA). Residual FAC levels in the biocide tank and WCT basins were measured using the diethyl-p-phenylene diamine-based colorimetric assay on a DR300 chlorine pocket colorimeter (HACH, USA) according to manufacturer’s instructions.

### Flow cytometry

Flow cytometry was used to enumerate the intact and membrane-compromised proportions of planktonic populations in the sampled waters. Intermediate stock solutions of 100 × SYBR Green I (SG, Sigma Aldrich, UK) and 600 µM propidium iodide (PI, Sigma Aldrich, UK) were prepared from master stocks by dilution in 10 mM TRIS (pH 8.1). 5 µL of SG or PI intermediate stocks were added to 495µL of sample, to bring final stain concentrations to 1 × and 6 µM (for SG and PI, respectively). For unstained control samples 5 µL of 0.85% w/v NaCl (sterile filtered) was used in place of SG or PI, to bring to the same final volume as stained subsamples (500 µL). Samples were briefly vortexed and incubated at 30 °C in the dark for 15 min with gentle mixing on an orbital shaker (at 110 RPM). Once stained, samples were immediately queued for acquisition on an Accuri C6 + (BD, USA). During acquisition a threshold of 12,000 on FSC-H was used, based on initial optimization tests using *E. coli* and earlier site samples. Samples were acquired using the blue laser line (488 nm), a flow rate of 15 µL/min, for accurate enumeration, with a core size of 16 µm and a sample volume limit of 25 µL. SG fluorescence was captured in FL1-H (533/30 nm) and PI was captured in FL3-H (670 LP). For this study, samples were not dual stained as we observed substantial spill over of SG signal into the FL3-H channel (where PI data was recorded) we therefore ran preliminary tests to ensure that proper inferences could be made when staining was separated across two subsamples (Supplementary Fig. S3). Counts remained below a maximum permitted density of 4000 events/µL, therefore no sample dilution was necessary.

### Biocide assay

A flow cytometric assay was carried out to evaluate the efficacy of oxidative biocides used at site and to establish whether resident biofouling communities exhibited resistance to the treatment. Water samples, collected from KC WCT basins were treated with a 10% FAC sodium hypochlorite solution (final assay concentration of 1 ppm FAC) obtained from the DCP operators, or left untreated. Samples were incubated at 30 °C for 15-min intervals over a 1 h period. After the allotted time, samples were stained with either SG or PI and subsequently analysed by FCM (as described in section "Flow cytometry").

### Data analysis

#### Computational flow cytometry

**Figure 7:**
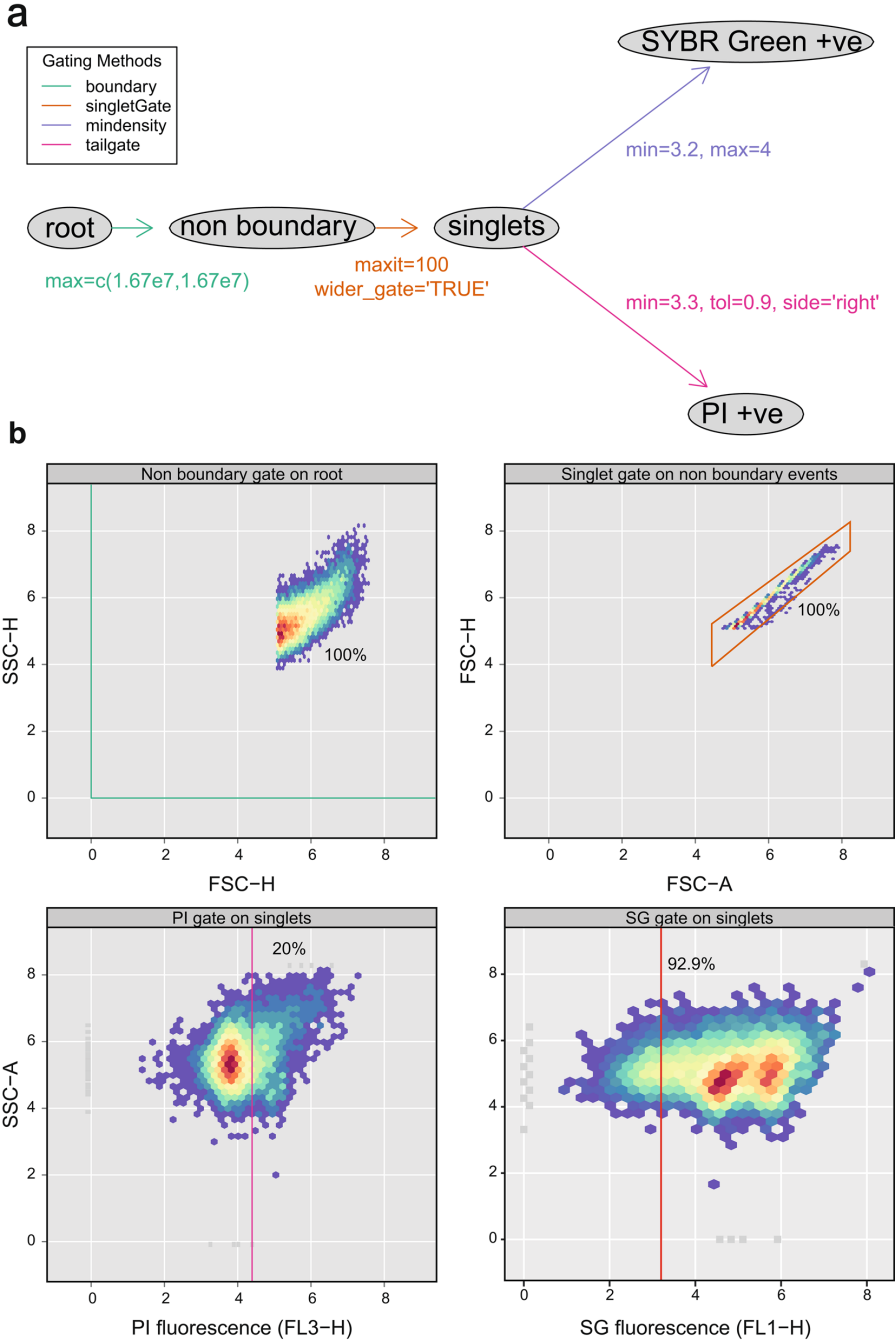
Gating strategy used for the study. **a**—Gating template showing the population nodes, gating methods and parameters used to calculate subpopulations. Where root is the starting population (all events), non-boundary population are those events which do not fall on the axes (the position of the boundary filter has been indicated as a green line in the plot). Singlets are those events within the singlet gate, SYBR Green + ve and PI + ve are those events which were positively gated in FL1-H and FL3-H, respectively. The min and max parameters for the SG gate are set to avoid erroneously gating between low and high nucleic acid content microbes. **b**—Example plots (based on a randomly selected PI-stained sample for the FL3-H plot and an SG-stained sample for the FL1-H plot) showing the gates used at each step (color-coded as in **a**). Autofluorescent events were observed for ~ 1–5% of the populations in the FL1-H and FL3-H channels (as shown in Supplementary Fig. S5).

Flow cytometry datasets (FCS files) were read into RStudio using the FlowCore package^31^. Flowset expression data (the per event readings in each of the cytometer channels) were transformed by means of the logicle transformation method in FlowCore having calculated the transformation parameters with the estimateLogicle function. Population statistics were then calculated using a user-defined gating template (Fig. 7a) for computational gating as part of the OpenCyto package^32^. Briefly, boundary events were removed with a boundary filter set to 1.67 × 10^7^ on each of the physical channels (SSC-H and FSC-H) which was based on the cytometer that was used (Accuri C6 Plus). Non-singlet events were removed by gating on FSC-H vs FSC-A using the singletGate function (with 100 iterations for the fit and the wider_gate option set to “TRUE” to accommodate variations from these environmental samples). SG-positive events (total stained cells (viable and membrane-compromised), were identified using the mindensity gate function (with a gating window between 3.2 and 4 on FL1-H) and PI-positive events (membrane-compromised cells) were identified using the tailgate function (with the tolerance set to 0.9 and the right-hand tail targeted) due to the relatively dim signal from PI in FL3-H (Fig. 7b). Flow cytometric plots were generated using the ggcyto, flowStats and flowViz packages ^33–35^. Gated population data (counts and proportions) were extracted from the gated data using OpenCyto. It was necessary to correct for differences in total event counts between subsample pairs (i.e. SG and PI stained counterparts) due to our choice of single staining over conventional dual staining. Intact cell counts (ICC) were calculated using the formulae described in Supplementary Methods.

As the study was focussed on enumerating microbial loads in these DCP systems, ICCs were used for comparative analyses (unless otherwise stated).

Illustrative examples of these calculations are provided in Supplementary Fig. S4, where further information on the formula used can be found.

An R script for the cFCM analytical pipeline used for this study can be found as a supplementary appendix to this article.

### Statistical analyses

Statistical analyses were carried out on R^36^ within RStudio^37^. Linear regressions and their ANOVA were carried out using the stats package in R. Nonparametric comparisons in group means were carried out using the Kruskal–Wallis test and, where pair-wise comparisons were required, a post-hoc Dunn’s test based on Benjamini–Hochberg correction was used in R as implemented in the FSA package^38^.

## Supplementary information

Supplementary Information.

## Data Availability

The flow cytometric dataset generated during the study, alongside all other water chemistry data and plant metadata, are available at the Mendeley Data repository (https://data.mendeley.com/datasets/998fpswh84/3). The R script and additional information can be found in the Supplementary Information files.

## References

[1] Eveloy V, Ayou DS (2019). Sustainable district cooling systems: status, challenges, and future opportunities, with emphasis on cooling-dominated regions. Energies.

[2] Olama AA (2016). District Cooling: Theory and Practice.

[3] Dakheel J, Tabet Aoul K, Hassan A (2018). Enhancing green building rating of a school under the hot climate of UAE; renewable energy application and system integration. Energies.

[4] Sarraf, G., Fayad, W., Sayed, T. E. & Monette, S.-P. Unlocking the Potential of District Cooling The Need for GCC Governments to Take Action. (Booz & Co. (now Strategy&), 2012).

[5] Gang W, Wang S, Xiao F, Gao DC (2016). District cooling systems: technology integration, system optimization, challenges and opportunities for applications. Renew. Sustain. Energy Rev..

[6] Liu F (2013). Optimizations of inhibitors compounding and applied conditions in simulated circulating cooling water system. Desalination.

[7] Liu Y (2009). Role of bacterial adhesion in the microbial ecology of biofilms in cooling tower systems. Biofouling.

[8] Di Pippo F, Di Gregorio L, Congestri R, Tandoi V, Rossetti S (2018). Biofilm growth and control in cooling water industrial systems. FEMS Microbiol. Ecol..

[9] Meesters KPH, Van Groenestijn JW, Gerritse J (2003). Biofouling reduction in recirculating cooling systems through biofiltration of process water. Water Res..

[10] Safford HR, Bischel HN (2019). Flow cytometry applications in water treatment, distribution, and reuse: a review. Water Res.

[11] Abraham PE, Giannone RJ, Xiong W, Hettich RL (2014). Metaproteomics: extracting and mining proteome information to characterize metabolic activities in microbial communities. Current Protocols Bioinform.

[12] Buysschaert B, Vermijs L, Naka A, Boon N, De Gusseme B (2018). Online flow cytometric monitoring of microbial water quality in a full-scale water treatment plant. npj Clean Water.

[13] Helmi K, David F, Di Martino P, Jaffrezic M-P, Ingrand V (2018). Assessment of flow cytometry for microbial water quality monitoring in cooling tower water and oxidizing biocide treatment efficiency. J. Microbiol. Methods.

[14] Van Nevel S (2017). Flow cytometry for immediate follow-up of drinking water networks after maintenance. Water Res..

[15] Park JW, Lee YJ, Meyer AS, Douterelo I, Maeng SK (2018). Bacterial growth through microfiltration membranes and NOM characteristics in an MF-RO integrated membrane system: Lab-scale and full-scale studies. Water Res..

[16] Hammes F, Egli T (2010). Cytometric methods for measuring bacteria in water: advantages, pitfalls and applications. Anal. Bioanal. Chem..

[17] Saeys Y, Van Gassen S, Lambrecht BN (2016). Computational flow cytometry: helping to make sense of high-dimensional immunology data. Nat. Rev. Immunol..

[18] Ling L (2012). Carboxylate-terminated double-hydrophilic block copolymer as an effective and environmental inhibitor in cooling water systems. Desalination.

[19] Zhu Z (2014). Effects of pipe materials on chlorine-resistant biofilm formation under long-term high chlorine level. Appl. Biochem. Biotechnol..

[20] Hammes F (2008). Flow-cytometric total bacterial cell counts as a descriptive microbiological parameter for drinking water treatment processes. Water Res..

[21] Van Nevel S (2017). Flow cytometric bacterial cell counts challenge conventional heterotrophic plate counts for routine microbiological drinking water monitoring. Water Res..

[22] Ding W (2019). Ozone disinfection of chlorine-resistant bacteria in drinking water. Water Res..

[23] Gillespie S (2014). Assessing microbiological water quality in drinking water distribution systems with disinfectant residual using flow cytometry. Water Res..

[24] Pinel ISM, Moed DH, Vrouwenvelder JS, van Loosdrecht MCM (2020). Bacterial community dynamics and disinfection impact in cooling water systems. Water Res..

[25] Mah TFC, O'Toole GA (2001). Mechanisms of biofilm resistance to antimicrobial agents. Trends Microbiol..

[26] Simões LC (2011). Persister cells in a biofilm treated with a biocide. Biofouling.

[27] Campana R, Ciandrini E, Baffone W (2018). Experimental approach for a possible integrated protocol to determine sanitizer activity against both planktonic bacteria and related biofilms. Food Res. Int..

[28] Larimer C (2016). A method for rapid quantitative assessment of biofilms with biomolecular staining and image analysis. Anal. Bioanal. Chem..

[29] Wilson, C. *et al.* Quantitative and Qualitative Assessment Methods for Biofilm Growth: A Mini-review. *Res Rev J Eng Technol* **6**. https://www.rroij.com/open-access/quantitative-and-qualitative-assessment-methods-for-biofilm-growth-a-minireview-.pdf (2017). PMC6133255 30214915

[30] Besmer MD (2014). The feasibility of automated online flow cytometry for in-situ monitoring of microbial dynamics in aquatic ecosystems. Frontiers Microbiol..

[31] Hahne F (2009). flowCore: a Bioconductor package for high throughput flow cytometry. BMC Bioinform..

[32] Finak G (2014). OpenCyto: An Open Source Infrastructure for Scalable, Robust, Reproducible, and Automated, End-to-End Flow Cytometry Data Analysis. PLoS Comput. Biol..

[33] Hahne, F., Gopalakrishnan, N., Khodabakhshi, A. H., Wong, C. & Lee, K. flowStats: Statistical methods for the analysis of flow cytometry data. *R Packag. version* 3 (2009).

[34] Sarkar D, Le Meur N, Gentleman R (2008). Using flowViz to visualize flow cytometry data. Bioinformatics.

[35] Van P, Jiang W, Gottardo R, Finak G (2018). ggCyto: next generation open-source visualization software for cytometry. Bioinformatics.

[36] R: A language and environment for statistical computing. R Foundation for Statistical Computing (Vienna, Austria, 2017).

[37] RStudio: Integrated Development for R. (RStudio, Inc., Boston, MA 2015).

[38] FSA: Fisheries Stock Analysis (2019).

