# Supplementary material for: Computational flow cytometry of planktonic populations for the evaluation of microbiological-control programs in district cooling plants
Source: Sci Rep. 2020 Aug 6;10:13299. doi: 10.1038/s41598-020-70198-5 (PMC7411017; doi:10.1038/s41598-020-70198-5)
Supplement: Supplementary file 1 — Supplementary Information. [file 41598_2020_70198_MOESM1_ESM.pdf]

Computational flow cytometry of planktonic populations for the evaluation of microbiological-control programs in district cooling plants

J.M.W.R. McElhinney[1], A. Mawart[1], R.S.S.M Alkaabi[1], H.S.S. Abdelsamad[1], A.M. Mansour[1] and A. Hasan*[1,2]

[1]. Applied Genomics Laboratory, Department of Biomedical Engineering, Khalifa University, Abu Dhabi, UAE

[2] Centre for Membranes and Advanced Water Technology (CMAT), Khalifa University, Abu Dhabi, UAE

*** Corresponding Author**:

Ayesha Hasan

Applied Genomics Laboratory

Building 1A, 2st Floor

Khalifa University, Masdar Campus

Masdar City

Abu Dhabi, Po Box 54224

United Arab Emirates

Ayesha.almarzooqi@ku.ac.ae

Supplementary information

Supplementary Methods

The following formulae were used to calculate the intact cell counts (ICCs). These were used to account for differences in total cell counts across paired subsamples (i.e. PI and SG stained counterpart samples) as described in Supplementary Fig. S4. Each step is detailed alongside an example for a typical 25µL C site (condenser outlet) sample with the following data

PI positive count in PI stained sample ($C_{pi} \subset S_{pi}$)	5,064
Total event count in PI stained sample ($C_T \subset S_{pi}$)	20,647
SG positive count in SG stained sample ($C_{sg} \subset S_{sg}$)	20,856
Total event count in SG stained sample ($C_T \subset S_{sg}$)	21,639

First, the estimated proportion of membrane-compromised cells ($Prop_{mcc}$) are computed. As differences in total event counts (C_T) between subsample pairs must be accounted for, $Prop_{mcc}$ is determined as the ratio between the proportion of PI-positive events over the proportion of SG-positive events. For this, the following formula (color-coded according to S.Fig.4) is used as presented in equation (1).

(1):

$$Prop_{mcc} = \frac{\left(\frac{C_{pi} \subset S_{pi}}{C_T \subset S_{pi}}\right)}{\left(\frac{C_{sg} \subset S_{sg}}{C_T \subset S_{sg}}\right)} \qquad \mathrm{Prop}_{mcc} = \frac{\left(\frac{5{,}064}{20{,}647}\right)}{\left(\frac{20{,}856}{21{,}639}\right)} = 0.2545$$

Where:

$C_{pi} \subset S_{pi}$ is the count of PI-positive events (C_{pi}) of a PI-stained sample (S_{pi}), $C_T \subset S_{pi}$ is the total event count (C_T) of S_{pi}.

$C_{sg} \subset S_{sg}$ is the count of SG-positive events (C_{sg}) of a SG-stained sample (S_{sg}), $C_T \subset S_{sg}$ is the total event count (C_T) of S_{sg}.

Second, the estimated proportion of intact cells ($Prop_{icc}$) is determined as a remainder of the total cell population (i.e. 1) following the subtraction of the proportion of membrane-compromised cells $Prop_{mcc}$ as shown in equation (2).

(2):

$$Prop_{icc} = 1 - Prop_{mcc} \qquad Prop_{icc} = 1 - 0.2545 = 0.7455$$

Third, the intact cell count (*ICC*) can then be determined as a factor of the estimated proportion of intact cells in samples multiplied by the count of SG-positive events in an SG-stained sample, $C_{sg} \subset S_{sg}$ as shown in equation (3).

(3):

$$ICC = C_{sg} \subset S_{sg} \times Prop_{icc} \qquad ICC = 20{,}856 \times 0.7455 = 15{,}549$$

As the analysed volume of sample was 25µL, the ICCs were then multiplied by 40 to adjust microbial populations to conventional per mL cell counts.

Supplementary Results

Quantitatively relating microbial loads to DCP performances is complicated by confounding factors and may require long term monitoring

Whilst biofouling is a well-known issue for DCPs[1-3], quantitative analyses of its impact on plant performance are limited in the literature. We therefore sought to assess whether short-term ICCs could inform on biofouling-related impacts on plant performance metrics from the SCADA systems at site.

The relationships between ICCs and chiller-level performance metrics (kW/TR, approach temperature and ΔT) were also examined (data not shown). However, no significant relationships could be identified in the time frame examined due to the low number of data points per chiller (chillers are frequently switched to maximise lifespan). A longer follow up of at least one complete cycle between deep clean events (~6 months) may provide enough data points for each chiller to draw such conclusions. We therefore focussed on plant-scale electrical and water performance metrics to build regression models against ICCs (Supplementary Fig. S6). ICCs were not a predictor of plant water performance for this study. For RB and YI, electrical performance was also not related to ICC levels. Given that RB is suspected of having relatively high calcium carbonate levels (Section 2.2) and low ICCs, any performance issues at RB are likely to be driven by scaling. However, ICCs from

KC open loop samples (sites B or C) were found to show statistically significant association with electrical plant performance (P <0.001). Since the MUW was found to be considerably cleaner than the open loop water (Fig. 3 and Supplementary Table 1), the extensive use of this clean water can be expected to reduce observed cell counts through dilution effects, whereas blowdown will remove cells from the system. It is therefore likely that this association between ICC and electrical performance at KC was observable as a result of the absence of blowdown and limited use of MUW (relative to the other plants) during the sampling program. Indeed, when MUW consumption and blowdown are plotted against ICCs there appears to be a clear increase in ICCs in both the WTC basin and condenser outlets when MUW consumption and blowdown decrease (Supplementary Fig. S4). However, it should be noted that this observation was not confirmed statistically. Nevertheless, it is clear that MUW use should be considered when monitoring biofouling populations in similar recirculating systems. Importantly, several well established factors for plant performance, including, approach temperature, chiller delta temperatures, TIC, TOC, conductivity and chiller performances also showed little or no correlation with plant performances overall during the study period. We suspect that this highlights the interplay of factors that ultimately give rise to plant performance overall. The potential interaction between multiple factors on observed ICCs and performance metrics, does raise a challenge for quantitatively relating biofouling to plant operations using planktonic counterparts in the community. Therefore, it is likely necessary to build up a long-term history of ICC readings at site in order to uncover the quantitative relationships between microbial loads and plant performances.

Given that YI was observed to have the most extensive biofouling in the WCT basins, and also used a lower frequency of biocide pulses per day (Table 1), the lower cell counts (relative to KC) was an unexpected finding (Fig. 3b). However, this plant used considerably more water than RB or KC. Therefore, at plants where the MUW consumption and blowdown volumes are high (such as YI) these operational practices can be expected to help in the control of microbial loads within the system, but at the cost of decreasing water performance of the plant (Fig. 2a). Whereas, at KC, the water usage is lower, and the levels of ICCs are primarily driven by biocide dosing and deep cleaning (Sections 3.4.2 and 3.4.3).

Supplementary Table

Supplementary Table 1 | Cooling water chemistry across plants[1]

Parameter	KC			RB			YI		
	A	B	C	A	B	C	A	B	C
Br (ppm)	n.d	n.d	n.d	n.d	n.d	n.d	n.d	n.d	n.d
Ca (ppm)	***3.08 ±0.2***	***15.23 ±10.1***	***24.73 ±4.2***	***3.28 ±0.8***	***23.92 ±3.8***	***24.38 ±5.0***	***2.23 ±0.8***	***13.05 ±6.2***	***15.98 ±5.3***
Cl (ppm)	***0.05 ±0.1***	***1.74 ±1.4***	***3.12 ±0.7***	***0.35 ±0.5***	***1.78 ±0.6***	***2.25 ±0.9***	***2.88 ±3.9***	***8.06 ±4.6***	***8.93 ±3.7***
Fl (ppm)	0.03 ±0.1	0.02 ±0.1	0.00 ±0.0	0.00 ±0.0	0.00 ±0.0	0.01 ±0.0	n.d	n.d	n.d
K (ppm)	***0.00 ±0.0***	***1.13 ±1.1***	***2.12 ±0.5***	***0.00 ±0.0***	***0.57 ±0.5***	***0.76 ±0.6***	***0.66 ±0.5***	***9.49 ±5.6***	***11.80 ±5.4***
Li (ppm)	0.00 ±0.0	0.00 ±0.0	0.00 ± 0.0	0.00 ±0.0	0.00 ±0.0	0.00 ±0.0	0.00 ±0.0	0.00 ±0.0	0.00 ±0.0
Mg (ppm)	***0.42 ±0.5***	***0.43 ±0.1***	*0.52 ±0.1*	**2.22 ±2.7**	**1.27 ±1.0**	2.17 ±3.4	**4.25 ±6.6**	**1.52 ±2.0**	1.47 ±2.2
Na (ppm)	***0.15 ±0.2***	***2.02 ±2.9***	***4.29 ±4.3***	***0.61 ±0.5***	***7.98 ±2.2***	***8.60 ±2.7***	***0.93 ±1.4***	***5.36 ±2.8***	***6.64 ±4.3***
NH_4 (ppm)	0.03 ±0.0	**0.05 ±0.0**	**0.05 ±0.0**	0.04 ±0.0	**0.06 ±0.0**	**0.05 ±0.0**	0.02 ±0.0	**0.03 ±0.0**	**0.02 ±0.0**
NO_2 (ppm)	n.d	n.d	n.d	n.d	n.d	n.d	n.d	n.d	n.d
NO_3 (ppm)	n.d	**1.79 ±1.6**	**3.22 ±0.6**	n.d	**1.19 ±0.3**	**1.23 ±0.4**	n.d	**0.41 ±0.4**	**0.48 ±0.4**
PO_4 (ppm)	n.d	n.d	n.d	n.d	n.d	n.d	n.d	n.d	n.d
SO_4 (ppm)	n.d	***1.32 ±1.8***	*2.74 ±2.1*	***0.05 ±0.2***	***3.07 ±0.9***	*3.34 ±1.2*	***1.01 ±2.0***	***2.11 ±2.7***	*2.34 ±2.3*
TOC (ppm)	***0.43 ±0.49***	**4.42 ±3.49**	**5.40 ±3.77**	***0.89 ±1.69***	**9.87 ±2.39**	**9.24 ±1.90**	***0.88 ±0.90***	**8.11 ±4.32**	**9.69 ±5.10**
TIC (ppm)	*16.14 ±0.51*	**71.83 ±30.48**	**81.38 ±33.50**	*16.30 ±2.44*	**99.04 ±8.40**	**100.94 ±13.70**	***12.31 ±1.23***	**58.46 ±17.04**	**58.53 ±17.91**
Conductivity (µS/cm)	***181 ±128***	930 ±477	**1140 ±376**	***145 ±24***	900 ±164	**932 ±143**	***357 ±146***	***1561 ±553***	***1861 ±260***
pH	*8.45 ±0.19*	8.97 ±0.34	8.97 ±0.34	*8.48 ±0.22*	9.05 ±0.23	9.08 ±0.21	***8.61 ±0.21***	**8.87 ±0.26**	**8.89 ±0.26**

[1] Data presented as the mean values obtained during the sampling period (± S.D). Significant differences in the means across sites (A, B, or C) are indicated in italics, whereas those with significant differences across plants (KC, RB or YI) are shown in bold. Significant differences are classified as those contrasts for which *P* <0.05, as determined by Kruskal-Walllis test. n.d – not detected (i.e. below detection limit).

Supplementary Figure Legends

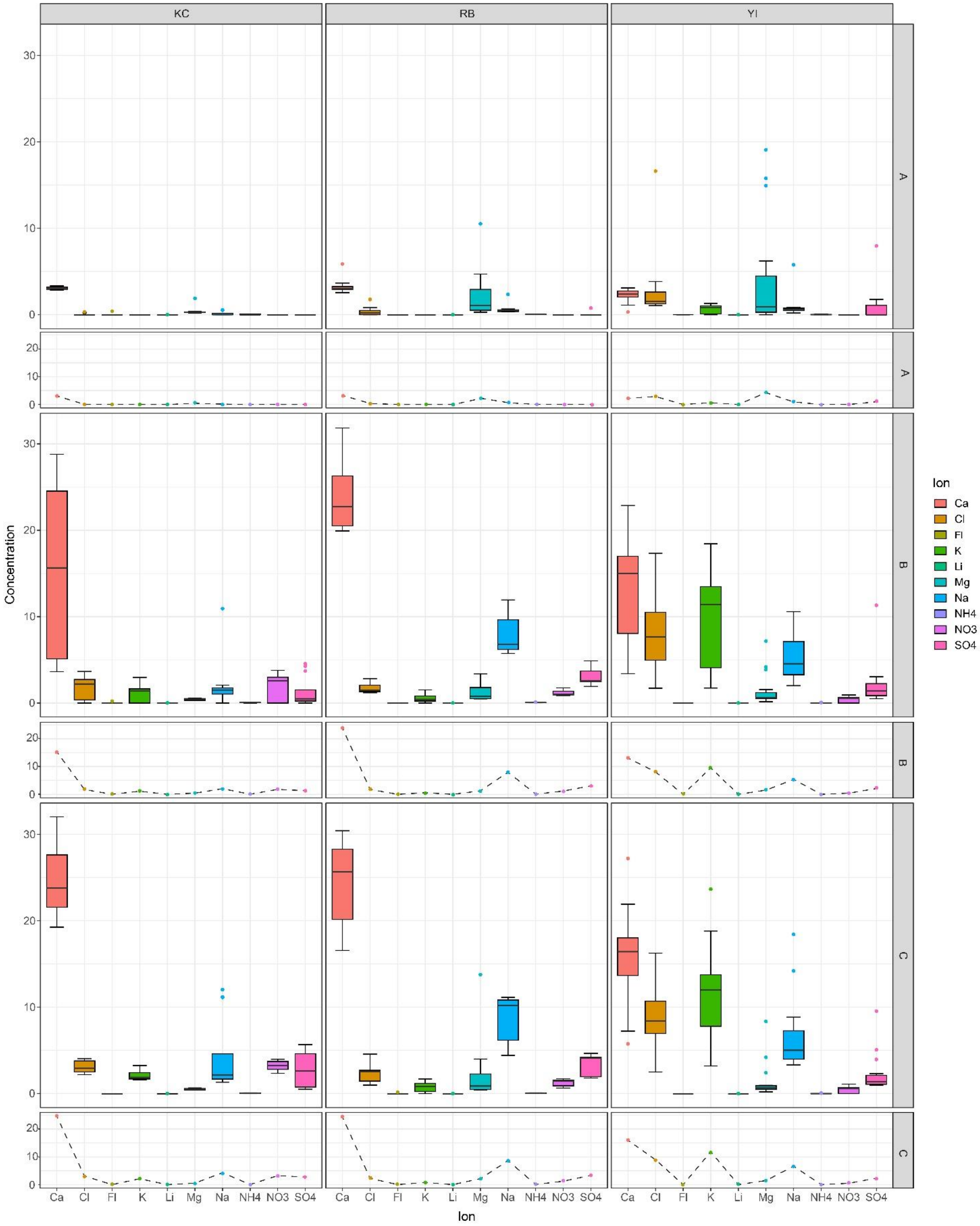

Supplementary Fig. S1 | Ionic water quality across the study sites shows high quality MUW and plant-specific declines in cooling water quality.

Data for each plant (KC, RB and YI) are segregated by column whereas data for sites (A, B and C) for each plant are segregated by row. Box boundaries represent the 25th (lower) and 75th (upper) percentiles (Q1 and Q3 respectively), upper whiskers show Q1 – 1.5 × interquartile range (IQR), lower whiskers show Q3 + 1.5 × IQR, data beyond those ranges are shown as individual points. A line plot is presented below each boxplot to highlight ionic profiles for each site per plant (wherein the line connects the mean values obtained for each ion at that site). Data for Br, NO_2 and PO_4 are omitted as these could not be detected at any point across the dataset. N= 126 (36 for KC, 45 for each of RB and YI).

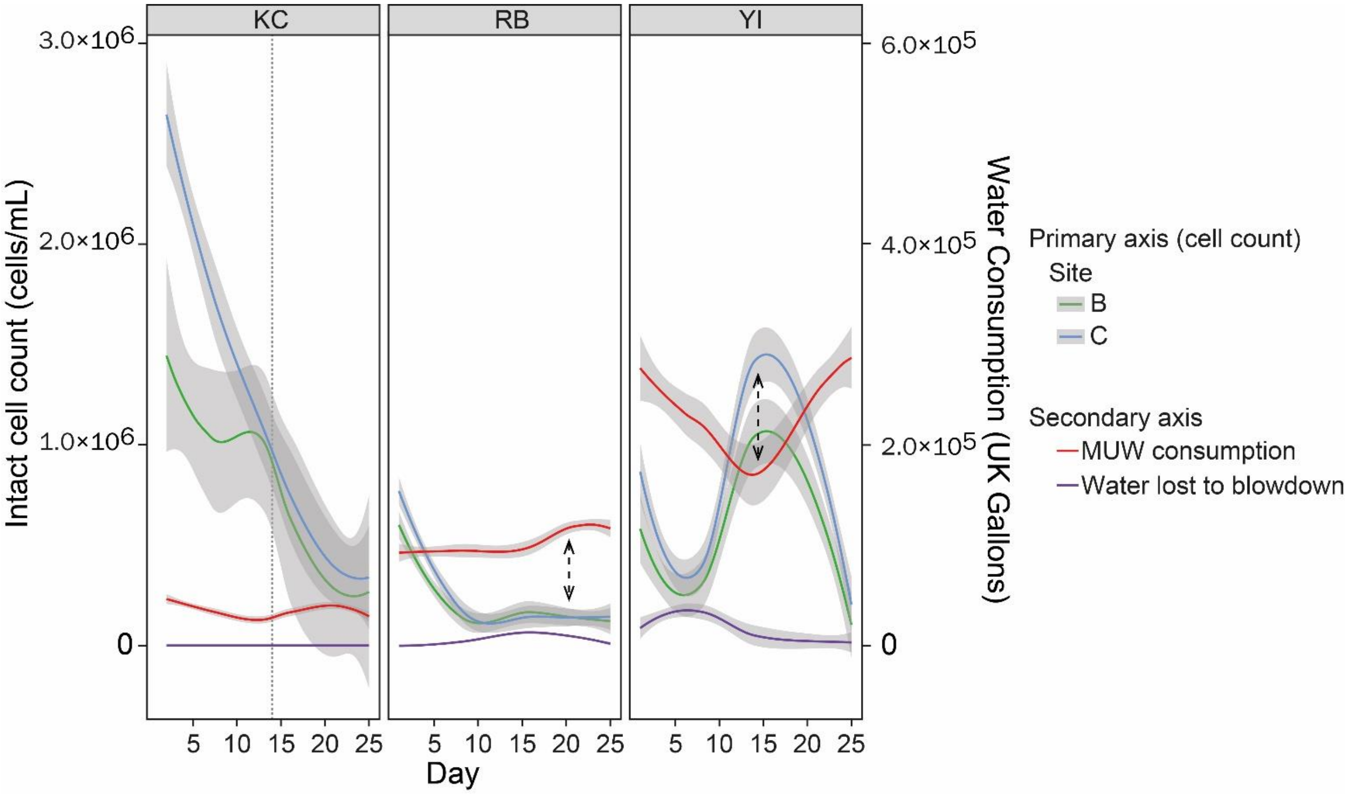

Supplementary Fig. S2 | Observed ICCs are influenced by operational water consumption

Plots of Loess fits and 95% confidence interval (grey) showing the relationship between daily make-up water (MUW) consumption (red), daily blowdown (purple) and cytometric intact cell counts (green (WCT basins) and blue (condenser outlets)), data points omitted for clarity. Dashed arrows highlight features where this relationship is clearest. Grey dotted line indicates the shift in biocide pulse frequency at plant KC.

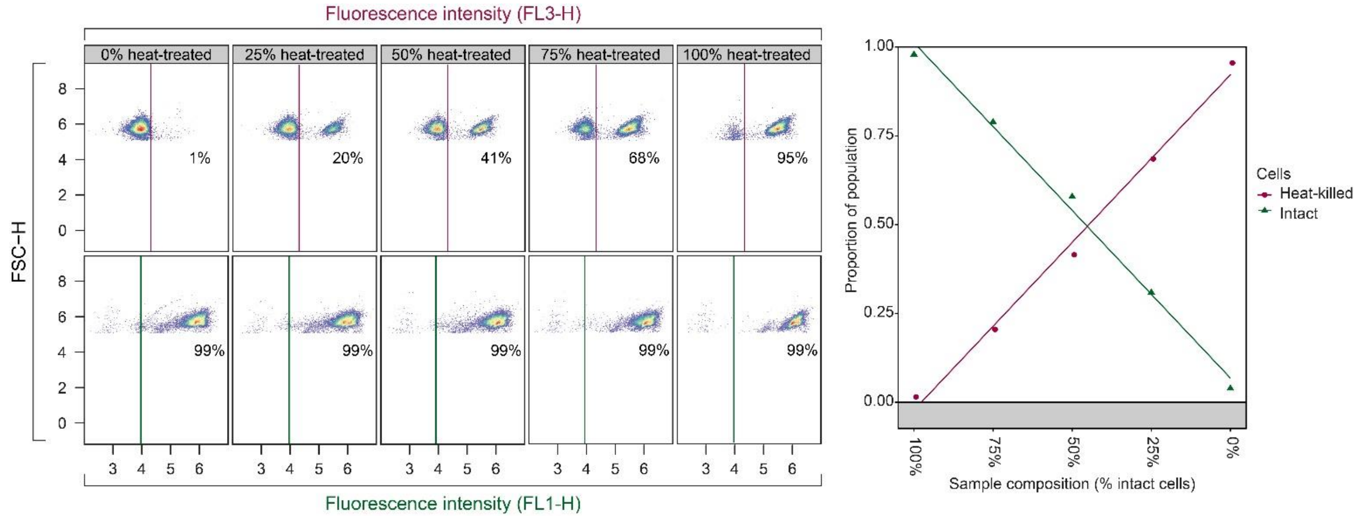

Supplementary Fig. S3 | Mock samples consisting of heat killed *E. coli* in known proportions with living cultures confirms reasonable results are obtained using computational cytometry on single stained samples.
Overnight *E. coli* DH5α cultures were spun down, diluted in 0.85% NaCl and subjected to heat-killing (80°C for 10 min). These heat-treated cultures were mixed v/v with untreated DH5α cultures (also suspended in 0.85% NaCl) at ratios of 100:0, 75:25, 50:50, 25:75 and 0:100 (untreated:treated). These mixed cell preparations were then stained as described in Section 2.4. Left panel – shows the computed gates for SG applied on FL1-H (bottom row) and PI, applied on FL3-H(top row) stained samples for each proportion of heat-killed cells, paired samples (i.e. those which have equivalent proportions of heat-killed cells) are vertically aligned. Intact cell counts were calculated as described in section **Error! Reference source not found.**). Here, *E. coli* was used as a representative Gram-negative bacteria (GNB), as GNB are known to be amongst the more recalcitrant microbes for SG-staining[4].

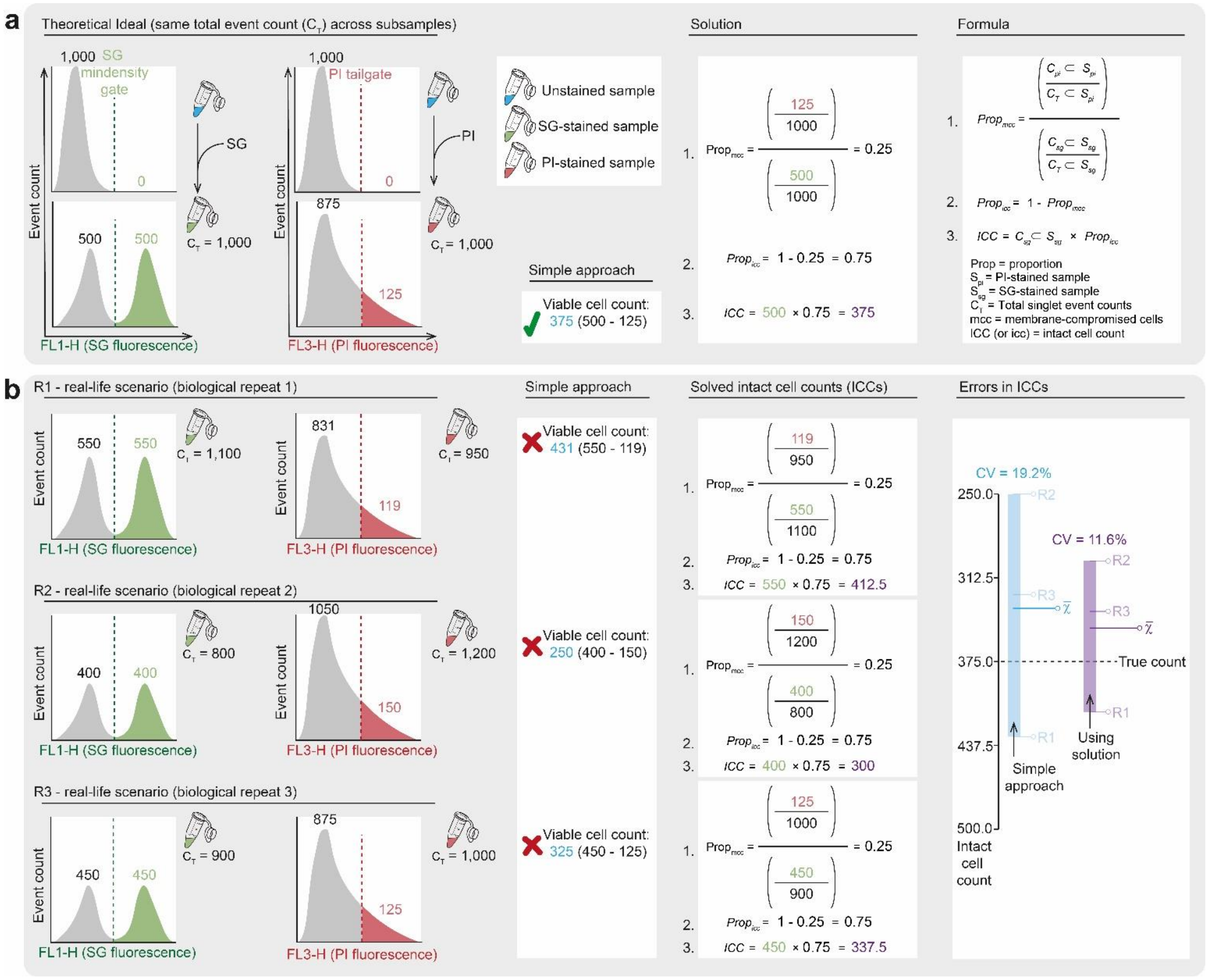

Supplementary Fig. S4 | Illustrative examples of how differences in total event count can be handled to arrive at reasonable approximations of viable cells when using a single staining approach for cell viability.

a – Theoretical ideal for a sample that has been analysed on the flow cytometer, either unstained, stained with SYBR green (SG) or stained with propidium iodide (PI). In this example, the sample possesses the following true characteristics (the unknowns that we seek to determine through the analysis): 1,000 events per 25 µL (the analysed volume), 50% of which are cells (500 cell events) of which 25% are non-viable membrane-compromised cells (125 cell events). In this theoretical ideal scenario, the total event counts (C_T) should equal 1,000 events for each subsample (unstained, SG-stained and PI-stained). The unstained subsample is used to generate a gate to define the events that are SG- or PI-positive (green and red dashed lines, respectively). Here, the SG-stained subsample should show 500 SG-negative events (for the 50% abiotic events in this example) and 500 events in the SG-positive region (for the 50% cells in this example). The PI-stained sample would show 875 events in the PI-negative region to the left of the gate (i.e. the 50% abiotic events plus 75% of the cell events which are intact and viable) and 125 events in the PI-positive region to the right of the gate (the 25% of cells, which are membrane-compromised). In this situation, the PI-positive non-viable cell count, can simply be subtracted from the SG-positive cell count to give the viable cells per 25 µL to arrive at the correct viable cell count. However, in reality, the stochastic nature of subsampling leads to variations in C_T between partner subsamples, which would lead to incorrect cell counts if PI-positive events were subtracted from SG-positive event counts if these differences are not accounted for. The 3 formula shown here were therefore used as a means to account for differences in C_T and a working example of these formula is given for this ideal scenario. **b** – A theoretical set of biological replicates of samples, with the same characteristics as the sample illustrated in **a**, but representing a real-life scenario where C_Ts differ between

subsamples. Here examples show severe differences in C_T and deviation from the expected C_T of 1,000 events. For these results, the ICC estimates are shown in blue (for the simple approach) and purple (using the formula presented in **a**). When the results of these estimates are plotted against the true count in this set of scenarios, we can see that the spread of the ICCs is roughly halved (CV of 11.6% vs 19.2%) and that the mean ($\bar{x}$) of the ICCs using these formula is a fairly good approximation (350, a 6.66% error) of the true ICC value (375) despite the high variance in C_T between subsamples. In this theoretical example, the CV between PI and SG stained sample readings is 22.1% whereas for the cooling tower dataset (with 414 paired SG and PI-stained subsamples) was 20.5%, we therefore expect the ICCs to be >94% accurate with this approach.

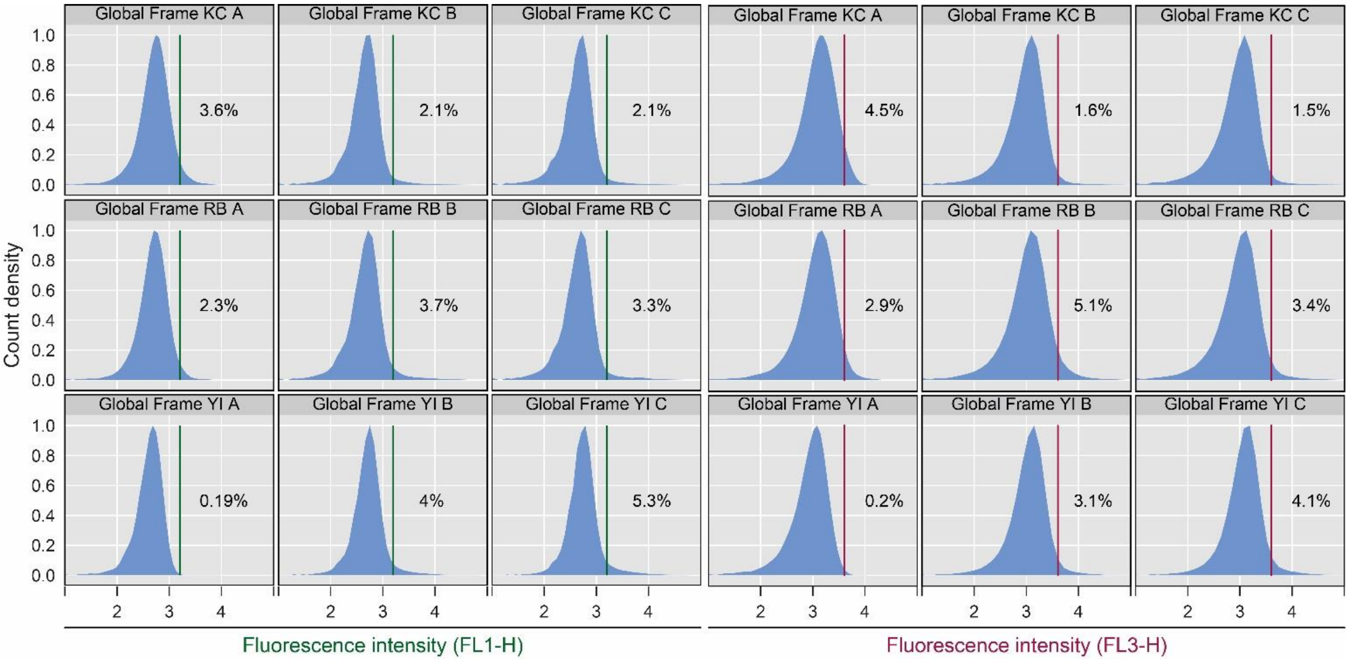

Supplementary Fig. S5 | Density histograms of representative frames for unstained samples indicating the results of computational gating in these control samples.

Green and red lines indicate the positions of the SG gate and PI gate (respectively). Percentages shown indicate the proportion of events that are SG or PI positive. Autofluorescence was observed for a small percentage of events (1-5%) which are taken to be the result of autofluorescent photosynthetic microbes in these waters.

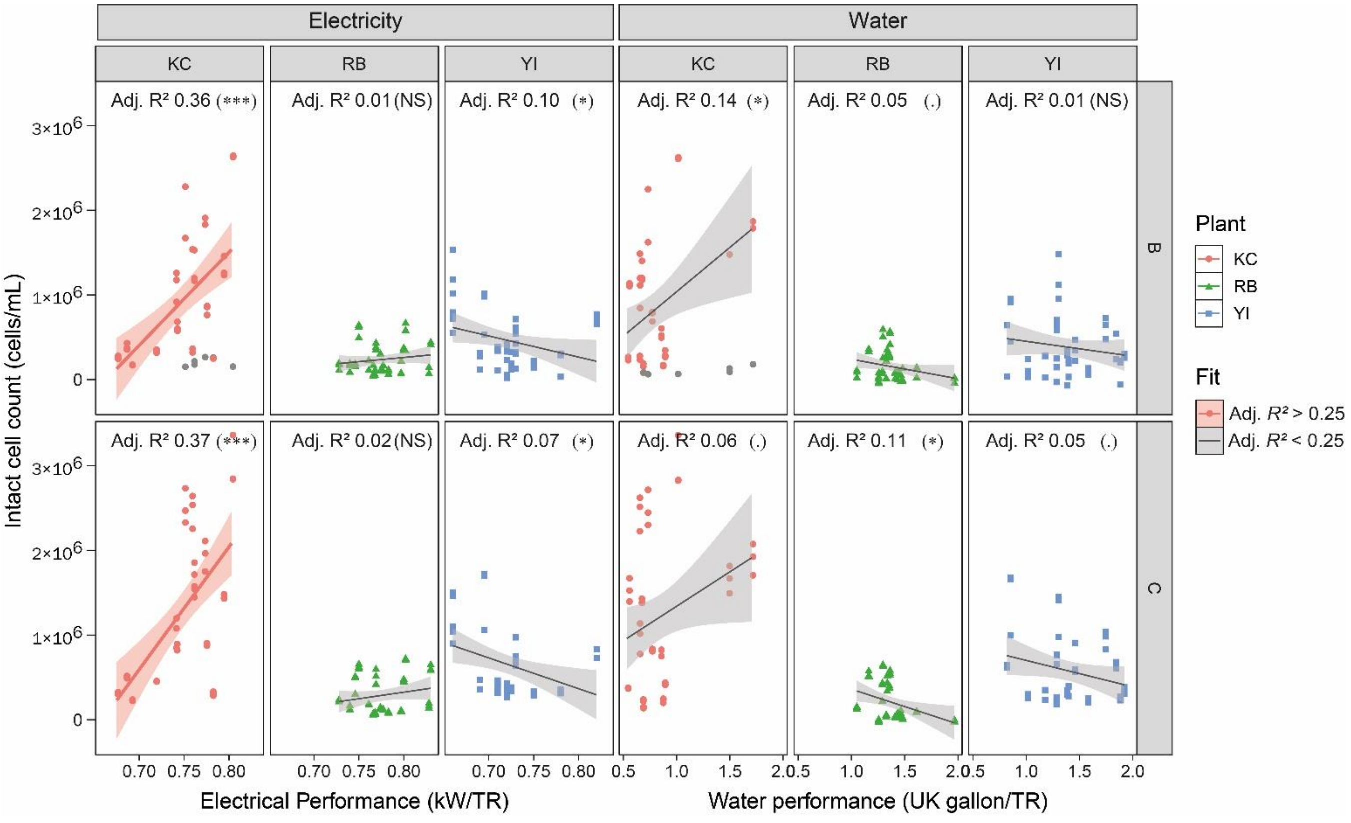

Supplementary Fig. S6 | ICCs in relation to DCP water and electrical performances.
Plant performances (electricity – left panel and water – right panel) are presented in relation to intact cell counts for samples from WCT basins (B sites) and condenser outlet waters (C sites). Linear regressions (showing the 95% confidence interval) are shown for each of the plant sites, those with an adjusted R^2 <0.2 are greyed out. Asterisks represent significance level where *** - $P < 0.001$, * - P <0.05 and "." – P <0.1. Data points are coloured according to plant except those in grey that represent those outlier KC samples, which were taken from WCT basins which had undergone deep cleaning but not been reconnected to the system (and were therefore excluded from the regression analyses).

Supplementary References

1 Di Pippo, F., Di Gregorio, L., Congestri, R., Tandoi, V. & Rossetti, S. Biofilm growth and control in cooling water industrial systems. *FEMS Microbiology Ecology* **94**, https://10.1093/femsec/fiy044 (2018).

2 Liu, Y. *et al.* Role of bacterial adhesion in the microbial ecology of biofilms in cooling tower systems. *Biofouling* **25**, 241-253, https://10.1080/08927010802713414 (2009).

3 Meesters, K. P. H., Van Groenestijn, J. W. & Gerritse, J. Biofouling reduction in recirculating cooling systems through biofiltration of process water. *Water Res* **37**, 525-532, https://10.1016/S0043-1354(02)00354-8 (2003).

4 Hammes, F. *et al.* Development and laboratory-scale testing of a fully automated online flow cytometer for drinking water analysis. *Cytometry Part A* **81A**, 508-516, https://10.1002/cyto.a.22048 (2012).

Supplementary Script

The following code can be used in R or RStudio to run the major processing and analysis for the cFCM used from the DCP water dataset. The code will first prepare the environment for analysis, process the .fcs files (containing the FCM data), apply the gating strategy, return key populations statistics and annotate cFCM data with site metadata. Some example plots along the way are given to allow the user to check for proper FCM data transformation and gating.

```
##### This code was written to accompany the article: Computational flow
cytometry of planktonic populations for the evaluation of
microbiological-control programs in district cooling plants, McElhinney,
et al. 2020 ##### Requires .fcs files as input (available from
https://data.mendeley.com/datasets/998fpswh84/1)
##### See session info below for full list of package versions, please
note that newer versions of ggcyto may require the ggcyto command to be
used in place of plotGate (code section 7.2), this will be evident if
plotGate returns empty graphs.
##### Requires plant data file "DCP metadata.csv" and a gating template
"gating_template.csv" (the template can be reproduced using the
information in Fig. 7a)
##### Paths need to be updated before running the code

##### 1 SET UP WORKSPACE #####
# 1.1 LOAD REQUIRED LIBRARIES #
library(flowCore)
library(flowStats)
library(flowViz)
library(flowWorkspace)
library(ggcyto)
library(openCyto)
library(ncdfFlow)

# 1.2 DEFINE QUICK CHANNEL NAMES #
fl1h<-"FL1-H"
fl2h<-"FL2-H"
fl3h<-"FL3-H"
fl4h<-"FL4-H"
fl1a<-"FL1-A"
fl2a<-"FL2-A"
fl3a<-"FL3-A"
fl4a<-"FL4-A"
fsca<-"FSC-A"
ssca<-"SSC-A"
ssch<-"SSC-H"
fsch<-"FSC-H"

mypathYI =" C:/../DCP_FCM_data/YI"
mypathRB =" C:/../DCP_FCM_data/RB"
mypathKC ="C:/../DCP_FCM_data/KC"


##### 2 IMPORT FLOW SETS #####

YIfs<-read.flowSet(path=mypathYI, pattern="*.fcs", as.is=T)
RBfs<-read.flowSet(path=mypathRB, pattern="*.fcs", as.is=T)
KCfs<-read.flowSet(path=mypathKC, pattern="*.fcs", as.is=T)

##### 3 IMPORT METADATA ####
```

```
##### 3.1 EXPORT PHENODATA TO CREATE BASIC METADATA FILES #####
#convert sample numbers to sample days
#retrieve sample numbers
#YI
YI.sample.nos<-substr(rownames(YIfs@phenoData@data), 14,16)
YI.sample.nos<-gsub("71-","071", YI.sample.nos)
YI.sample.nos<-gsub("72-","072", YI.sample.nos)
YI.sample.nos<-gsub("73-","073", YI.sample.nos)
YI.sample.nos<-gsub("81-","081", YI.sample.nos)
YI.sample.nos<-gsub("82-","082", YI.sample.nos)
YI.sample.nos<-gsub("83-","083", YI.sample.nos)
YI.sample.nos<-gsub("91-","091", YI.sample.nos)
YI.sample.nos<-gsub("92-","092", YI.sample.nos)
YI.sample.nos<-gsub("93-","093", YI.sample.nos)
YI.sample.nos
#RB
RB.sample.nos<-substr(rownames(RBfs@phenoData@data), 14,16)
RB.sample.nos<-gsub("71-","071", RB.sample.nos)
RB.sample.nos<-gsub("72-","072", RB.sample.nos)
RB.sample.nos<-gsub("73-","073", RB.sample.nos)
RB.sample.nos<-gsub("81-","081", RB.sample.nos)
RB.sample.nos<-gsub("82-","082", RB.sample.nos)
RB.sample.nos<-gsub("83-","083", RB.sample.nos)
RB.sample.nos<-gsub("91-","091", RB.sample.nos)
RB.sample.nos<-gsub("92-","092", RB.sample.nos)
RB.sample.nos<-gsub("93-","093", RB.sample.nos)
RB.sample.nos
#KC
KC.sample.nos<-substr(rownames(KCfs@phenoData@data), 14,16)
KC.sample.nos<-gsub("71-","071", KC.sample.nos)
KC.sample.nos<-gsub("72-","072", KC.sample.nos)
KC.sample.nos<-gsub("73-","073", KC.sample.nos)
KC.sample.nos<-gsub("81-","081", KC.sample.nos)
KC.sample.nos<-gsub("82-","082", KC.sample.nos)
KC.sample.nos<-gsub("83-","083", KC.sample.nos)
KC.sample.nos<-gsub("91-","091", KC.sample.nos)
KC.sample.nos<-gsub("92-","092", KC.sample.nos)
KC.sample.nos<-gsub("93-","093", KC.sample.nos)
KC.sample.nos
# convert to days
YI.days<-YI.sample.nos
YI.days<-ifelse(grepl("071", YI.days), 1, YI.days)
YI.days<-ifelse(grepl("072", YI.days), 1, YI.days)
YI.days<-ifelse(grepl("073", YI.days), 1, YI.days)
YI.days<-ifelse(grepl("081", YI.days), 2, YI.days)
YI.days<-ifelse(grepl("082", YI.days), 2, YI.days)
YI.days<-ifelse(grepl("083", YI.days), 2, YI.days)
YI.days<-ifelse(grepl("091", YI.days), 3, YI.days)
YI.days<-ifelse(grepl("092", YI.days), 3, YI.days)
YI.days<-ifelse(grepl("093", YI.days), 3, YI.days)
YI.days<-ifelse(grepl("101", YI.days), 4, YI.days)
YI.days<-ifelse(grepl("102", YI.days), 4, YI.days)
YI.days<-ifelse(grepl("103", YI.days), 4, YI.days)
YI.days<-ifelse(grepl("111", YI.days), 5, YI.days)
YI.days<-ifelse(grepl("112", YI.days), 5, YI.days)
YI.days<-ifelse(grepl("113", YI.days), 5, YI.days)
YI.days<-ifelse(grepl("121", YI.days), 6, YI.days)
YI.days<-ifelse(grepl("122", YI.days), 6, YI.days)
```

```
YI.days<-ifelse(grepl("123", YI.days), 6, YI.days)
YI.days<-ifelse(grepl("131", YI.days), 7, YI.days)
YI.days<-ifelse(grepl("132", YI.days), 7, YI.days)
YI.days<-ifelse(grepl("133", YI.days), 7, YI.days)
YI.days<-ifelse(grepl("141", YI.days), 8, YI.days)
YI.days<-ifelse(grepl("142", YI.days), 8, YI.days)
YI.days<-ifelse(grepl("143", YI.days), 8, YI.days)
YI.days<-ifelse(grepl("151", YI.days), 9, YI.days)
YI.days<-ifelse(grepl("152", YI.days), 9, YI.days)
YI.days<-ifelse(grepl("153", YI.days), 9, YI.days)
YI.days<-ifelse(grepl("161", YI.days), 10, YI.days)
YI.days<-ifelse(grepl("162", YI.days), 10, YI.days)
YI.days<-ifelse(grepl("163", YI.days), 10, YI.days)
YI.days<-ifelse(grepl("171", YI.days), 11, YI.days)
YI.days<-ifelse(grepl("172", YI.days), 11, YI.days)
YI.days<-ifelse(grepl("173", YI.days), 11, YI.days)
YI.days<-ifelse(grepl("181", YI.days), 12, YI.days)
YI.days<-ifelse(grepl("182", YI.days), 12, YI.days)
YI.days<-ifelse(grepl("183", YI.days), 12, YI.days)
YI.days<-ifelse(grepl("191", YI.days), 13, YI.days)
YI.days<-ifelse(grepl("192", YI.days), 13, YI.days)
YI.days<-ifelse(grepl("193", YI.days), 13, YI.days)
YI.days<-ifelse(grepl("201", YI.days), 14, YI.days)
YI.days<-ifelse(grepl("202", YI.days), 14, YI.days)
YI.days<-ifelse(grepl("203", YI.days), 14, YI.days)
YI.days<-ifelse(grepl("211", YI.days), 15, YI.days)
YI.days<-ifelse(grepl("212", YI.days), 15, YI.days)
YI.days<-ifelse(grepl("213", YI.days), 15, YI.days)
YI.days<-ifelse(grepl("221", YI.days), 16, YI.days)
YI.days<-ifelse(grepl("222", YI.days), 16, YI.days)
YI.days<-ifelse(grepl("223", YI.days), 16, YI.days)
YI.days<-ifelse(grepl("231", YI.days), 17, YI.days)
YI.days<-ifelse(grepl("232", YI.days), 17, YI.days)
YI.days<-ifelse(grepl("233", YI.days), 17, YI.days)
YI.days<-ifelse(grepl("241", YI.days), 18, YI.days)
YI.days<-ifelse(grepl("242", YI.days), 18, YI.days)
YI.days<-ifelse(grepl("243", YI.days), 18, YI.days)
YI.days<-ifelse(grepl("251", YI.days), 19, YI.days)
YI.days<-ifelse(grepl("252", YI.days), 19, YI.days)
YI.days<-ifelse(grepl("253", YI.days), 19, YI.days)
YI.days<-ifelse(grepl("261", YI.days), 20, YI.days)
YI.days<-ifelse(grepl("262", YI.days), 20, YI.days)
YI.days<-ifelse(grepl("263", YI.days), 20, YI.days)
YI.days<-ifelse(grepl("271", YI.days), 21, YI.days)
YI.days<-ifelse(grepl("272", YI.days), 21, YI.days)
YI.days<-ifelse(grepl("273", YI.days), 21, YI.days)
YI.days<-ifelse(grepl("281", YI.days), 22, YI.days)
YI.days<-ifelse(grepl("282", YI.days), 22, YI.days)
YI.days<-ifelse(grepl("283", YI.days), 22, YI.days)
YI.days<-ifelse(grepl("291", YI.days), 23, YI.days)
YI.days<-ifelse(grepl("292", YI.days), 23, YI.days)
YI.days<-ifelse(grepl("293", YI.days), 23, YI.days)
YI.days<-ifelse(grepl("301", YI.days), 24, YI.days)
YI.days<-ifelse(grepl("302", YI.days), 24, YI.days)
YI.days<-ifelse(grepl("303", YI.days), 24, YI.days)
YI.days<-ifelse(grepl("311", YI.days), 25, YI.days)
YI.days<-ifelse(grepl("312", YI.days), 25, YI.days)
YI.days<-ifelse(grepl("313", YI.days), 25, YI.days)
```

```
YI.days<-as.numeric(YI.days)
#RB
RB.days<-RB.sample.nos
RB.days<-ifelse(grepl("071", RB.days), 1, RB.days)
RB.days<-ifelse(grepl("072", RB.days), 1, RB.days)
RB.days<-ifelse(grepl("073", RB.days), 1, RB.days)
RB.days<-ifelse(grepl("081", RB.days), 2, RB.days)
RB.days<-ifelse(grepl("082", RB.days), 2, RB.days)
RB.days<-ifelse(grepl("083", RB.days), 2, RB.days)
RB.days<-ifelse(grepl("091", RB.days), 3, RB.days)
RB.days<-ifelse(grepl("092", RB.days), 3, RB.days)
RB.days<-ifelse(grepl("093", RB.days), 3, RB.days)
RB.days<-ifelse(grepl("101", RB.days), 4, RB.days)
RB.days<-ifelse(grepl("102", RB.days), 4, RB.days)
RB.days<-ifelse(grepl("103", RB.days), 4, RB.days)
RB.days<-ifelse(grepl("111", RB.days), 5, RB.days)
RB.days<-ifelse(grepl("112", RB.days), 5, RB.days)
RB.days<-ifelse(grepl("113", RB.days), 5, RB.days)
RB.days<-ifelse(grepl("121", RB.days), 6, RB.days)
RB.days<-ifelse(grepl("122", RB.days), 6, RB.days)
RB.days<-ifelse(grepl("123", RB.days), 6, RB.days)
RB.days<-ifelse(grepl("131", RB.days), 7, RB.days)
RB.days<-ifelse(grepl("132", RB.days), 7, RB.days)
RB.days<-ifelse(grepl("133", RB.days), 7, RB.days)
RB.days<-ifelse(grepl("141", RB.days), 8, RB.days)
RB.days<-ifelse(grepl("142", RB.days), 8, RB.days)
RB.days<-ifelse(grepl("143", RB.days), 8, RB.days)
RB.days<-ifelse(grepl("151", RB.days), 9, RB.days)
RB.days<-ifelse(grepl("152", RB.days), 9, RB.days)
RB.days<-ifelse(grepl("153", RB.days), 9, RB.days)
RB.days<-ifelse(grepl("161", RB.days), 10, RB.days)
RB.days<-ifelse(grepl("162", RB.days), 10, RB.days)
RB.days<-ifelse(grepl("163", RB.days), 10, RB.days)
RB.days<-ifelse(grepl("171", RB.days), 11, RB.days)
RB.days<-ifelse(grepl("172", RB.days), 11, RB.days)
RB.days<-ifelse(grepl("173", RB.days), 11, RB.days)
RB.days<-ifelse(grepl("181", RB.days), 12, RB.days)
RB.days<-ifelse(grepl("182", RB.days), 12, RB.days)
RB.days<-ifelse(grepl("183", RB.days), 12, RB.days)
RB.days<-ifelse(grepl("191", RB.days), 13, RB.days)
RB.days<-ifelse(grepl("192", RB.days), 13, RB.days)
RB.days<-ifelse(grepl("193", RB.days), 13, RB.days)
RB.days<-ifelse(grepl("201", RB.days), 14, RB.days)
RB.days<-ifelse(grepl("202", RB.days), 14, RB.days)
RB.days<-ifelse(grepl("203", RB.days), 14, RB.days)
RB.days<-ifelse(grepl("211", RB.days), 15, RB.days)
RB.days<-ifelse(grepl("212", RB.days), 15, RB.days)
RB.days<-ifelse(grepl("213", RB.days), 15, RB.days)
RB.days<-ifelse(grepl("221", RB.days), 16, RB.days)
RB.days<-ifelse(grepl("222", RB.days), 16, RB.days)
RB.days<-ifelse(grepl("223", RB.days), 16, RB.days)
RB.days<-ifelse(grepl("231", RB.days), 17, RB.days)
RB.days<-ifelse(grepl("232", RB.days), 17, RB.days)
RB.days<-ifelse(grepl("233", RB.days), 17, RB.days)
RB.days<-ifelse(grepl("241", RB.days), 18, RB.days)
RB.days<-ifelse(grepl("242", RB.days), 18, RB.days)
RB.days<-ifelse(grepl("243", RB.days), 18, RB.days)
RB.days<-ifelse(grepl("251", RB.days), 19, RB.days)
```

```
RB.days<-ifelse(grepl("252", RB.days), 19, RB.days)
RB.days<-ifelse(grepl("253", RB.days), 19, RB.days)
RB.days<-ifelse(grepl("261", RB.days), 20, RB.days)
RB.days<-ifelse(grepl("262", RB.days), 20, RB.days)
RB.days<-ifelse(grepl("263", RB.days), 20, RB.days)
RB.days<-ifelse(grepl("271", RB.days), 21, RB.days)
RB.days<-ifelse(grepl("272", RB.days), 21, RB.days)
RB.days<-ifelse(grepl("273", RB.days), 21, RB.days)
RB.days<-ifelse(grepl("281", RB.days), 22, RB.days)
RB.days<-ifelse(grepl("282", RB.days), 22, RB.days)
RB.days<-ifelse(grepl("283", RB.days), 22, RB.days)
RB.days<-ifelse(grepl("291", RB.days), 23, RB.days)
RB.days<-ifelse(grepl("292", RB.days), 23, RB.days)
RB.days<-ifelse(grepl("293", RB.days), 23, RB.days)
RB.days<-ifelse(grepl("301", RB.days), 24, RB.days)
RB.days<-ifelse(grepl("302", RB.days), 24, RB.days)
RB.days<-ifelse(grepl("303", RB.days), 24, RB.days)
RB.days<-ifelse(grepl("311", RB.days), 25, RB.days)
RB.days<-ifelse(grepl("312", RB.days), 25, RB.days)
RB.days<-ifelse(grepl("313", RB.days), 25, RB.days)
RB.days<-as.numeric(RB.days)
#KC
KC.days<-KC.sample.nos
KC.days<-ifelse(grepl("071", KC.days), 1, KC.days)
KC.days<-ifelse(grepl("072", KC.days), 1, KC.days)
KC.days<-ifelse(grepl("073", KC.days), 1, KC.days)
KC.days<-ifelse(grepl("081", KC.days), 2, KC.days)
KC.days<-ifelse(grepl("082", KC.days), 2, KC.days)
KC.days<-ifelse(grepl("083", KC.days), 2, KC.days)
KC.days<-ifelse(grepl("091", KC.days), 3, KC.days)
KC.days<-ifelse(grepl("092", KC.days), 3, KC.days)
KC.days<-ifelse(grepl("093", KC.days), 3, KC.days)
KC.days<-ifelse(grepl("101", KC.days), 4, KC.days)
KC.days<-ifelse(grepl("102", KC.days), 4, KC.days)
KC.days<-ifelse(grepl("103", KC.days), 4, KC.days)
KC.days<-ifelse(grepl("111", KC.days), 5, KC.days)
KC.days<-ifelse(grepl("112", KC.days), 5, KC.days)
KC.days<-ifelse(grepl("113", KC.days), 5, KC.days)
KC.days<-ifelse(grepl("121", KC.days), 6, KC.days)
KC.days<-ifelse(grepl("122", KC.days), 6, KC.days)
KC.days<-ifelse(grepl("123", KC.days), 6, KC.days)
KC.days<-ifelse(grepl("131", KC.days), 7, KC.days)
KC.days<-ifelse(grepl("132", KC.days), 7, KC.days)
KC.days<-ifelse(grepl("133", KC.days), 7, KC.days)
KC.days<-ifelse(grepl("141", KC.days), 8, KC.days)
KC.days<-ifelse(grepl("142", KC.days), 8, KC.days)
KC.days<-ifelse(grepl("143", KC.days), 8, KC.days)
KC.days<-ifelse(grepl("151", KC.days), 9, KC.days)
KC.days<-ifelse(grepl("152", KC.days), 9, KC.days)
KC.days<-ifelse(grepl("153", KC.days), 9, KC.days)
KC.days<-ifelse(grepl("161", KC.days), 10, KC.days)
KC.days<-ifelse(grepl("162", KC.days), 10, KC.days)
KC.days<-ifelse(grepl("163", KC.days), 10, KC.days)
KC.days<-ifelse(grepl("171", KC.days), 11, KC.days)
KC.days<-ifelse(grepl("172", KC.days), 11, KC.days)
KC.days<-ifelse(grepl("173", KC.days), 11, KC.days)
KC.days<-ifelse(grepl("181", KC.days), 12, KC.days)
KC.days<-ifelse(grepl("182", KC.days), 12, KC.days)
```

```
KC.days<-ifelse(grepl("183", KC.days), 12, KC.days)
KC.days<-ifelse(grepl("191", KC.days), 13, KC.days)
KC.days<-ifelse(grepl("192", KC.days), 13, KC.days)
KC.days<-ifelse(grepl("193", KC.days), 13, KC.days)
KC.days<-ifelse(grepl("201", KC.days), 14, KC.days)
KC.days<-ifelse(grepl("202", KC.days), 14, KC.days)
KC.days<-ifelse(grepl("203", KC.days), 14, KC.days)
KC.days<-ifelse(grepl("211", KC.days), 15, KC.days)
KC.days<-ifelse(grepl("212", KC.days), 15, KC.days)
KC.days<-ifelse(grepl("213", KC.days), 15, KC.days)
KC.days<-ifelse(grepl("221", KC.days), 16, KC.days)
KC.days<-ifelse(grepl("222", KC.days), 16, KC.days)
KC.days<-ifelse(grepl("223", KC.days), 16, KC.days)
KC.days<-ifelse(grepl("231", KC.days), 17, KC.days)
KC.days<-ifelse(grepl("232", KC.days), 17, KC.days)
KC.days<-ifelse(grepl("233", KC.days), 17, KC.days)
KC.days<-ifelse(grepl("241", KC.days), 18, KC.days)
KC.days<-ifelse(grepl("242", KC.days), 18, KC.days)
KC.days<-ifelse(grepl("243", KC.days), 18, KC.days)
KC.days<-ifelse(grepl("251", KC.days), 19, KC.days)
KC.days<-ifelse(grepl("252", KC.days), 19, KC.days)
KC.days<-ifelse(grepl("253", KC.days), 19, KC.days)
KC.days<-ifelse(grepl("261", KC.days), 20, KC.days)
KC.days<-ifelse(grepl("262", KC.days), 20, KC.days)
KC.days<-ifelse(grepl("263", KC.days), 20, KC.days)
KC.days<-ifelse(grepl("271", KC.days), 21, KC.days)
KC.days<-ifelse(grepl("272", KC.days), 21, KC.days)
KC.days<-ifelse(grepl("273", KC.days), 21, KC.days)
KC.days<-ifelse(grepl("281", KC.days), 22, KC.days)
KC.days<-ifelse(grepl("282", KC.days), 22, KC.days)
KC.days<-ifelse(grepl("283", KC.days), 22, KC.days)
KC.days<-ifelse(grepl("291", KC.days), 23, KC.days)
KC.days<-ifelse(grepl("292", KC.days), 23, KC.days)
KC.days<-ifelse(grepl("293", KC.days), 23, KC.days)
KC.days<-ifelse(grepl("301", KC.days), 24, KC.days)
KC.days<-ifelse(grepl("302", KC.days), 24, KC.days)
KC.days<-ifelse(grepl("303", KC.days), 24, KC.days)
KC.days<-ifelse(grepl("311", KC.days), 25, KC.days)
KC.days<-ifelse(grepl("312", KC.days), 25, KC.days)
KC.days<-ifelse(grepl("313", KC.days), 25, KC.days)
KC.days<-as.numeric(KC.days)
#retrieve other key metadata from sample info
#YI
YI.names<-rownames(YIfs@phenoData@data)
YI.sites<-substr(rownames(YIfs@phenoData@data), 8, 8)
YI.plant<-substr(rownames(YIfs@phenoData@data), 5, 6)
YI.stain<-substr(rownames(YIfs@phenoData@data), 18, 19)
YI.stain[1:160]<-gsub("S.","US", YI.stain[1:160])
YI.stain<-gsub("G.","SG", YI.stain)
YI.stain<-gsub("I.","PI", YI.stain)
YI.samples<-substr(rownames(YIfs@phenoData@data), 5, 16)
YI.samples<-gsub("71-","071", YI.samples)
YI.samples<-gsub("72-","072", YI.samples)
YI.samples<-gsub("73-","073", YI.samples)
YI.samples<-gsub("81-","081", YI.samples)
YI.samples<-gsub("82-","082", YI.samples)
YI.samples<-gsub("83-","083", YI.samples)
YI.samples<-gsub("91-","091", YI.samples)
```

```
YI.samples<-gsub("92-","092", YI.samples)
YI.samples<-gsub("93-","093", YI.samples)
#YI.sample.no<- substr(rownames(YIfs@phenoData@data), 14, 16)
#RB
RB.names<-rownames(RBfs@phenoData@data)
RB.sites<-substr(rownames(RBfs@phenoData@data), 8, 8)
RB.plant<-substr(rownames(RBfs@phenoData@data), 5, 6)
RB.stain<-substr(rownames(RBfs@phenoData@data), 18, 19)
RB.stain[1:160]<-gsub("S.","US", RB.stain[1:160])
RB.stain<-gsub("G.","SG", RB.stain)
RB.stain<-gsub("I.","PI", RB.stain)
RB.samples<-substr(rownames(RBfs@phenoData@data), 5, 16)
RB.samples<-gsub("71-","071", RB.samples)
RB.samples<-gsub("72-","072", RB.samples)
RB.samples<-gsub("73-","073", RB.samples)
RB.samples<-gsub("81-","081", RB.samples)
RB.samples<-gsub("82-","082", RB.samples)
RB.samples<-gsub("83-","083", RB.samples)
RB.samples<-gsub("91-","091", RB.samples)
RB.samples<-gsub("92-","092", RB.samples)
RB.samples<-gsub("93-","093", RB.samples)
#RB.sample.no<- substr(rownames(RBfs@phenoData@data), 14, 16)
#KC
KC.names<-rownames(KCfs@phenoData@data)
KC.sites<-substr(rownames(KCfs@phenoData@data), 8, 8)
KC.plant<-substr(rownames(KCfs@phenoData@data), 5, 6)
KC.stain<-substr(rownames(KCfs@phenoData@data), 18, 19)
KC.stain[1:160]<-gsub("S.","US", KC.stain[1:160])
KC.stain<-gsub("G.","SG", KC.stain)
KC.stain<-gsub("I.","PI", KC.stain)
KC.samples<-substr(rownames(KCfs@phenoData@data), 5, 16)
KC.samples<-gsub("71-","071", KC.samples)
KC.samples<-gsub("72-","072", KC.samples)
KC.samples<-gsub("73-","073", KC.samples)
KC.samples<-gsub("81-","081", KC.samples)
KC.samples<-gsub("82-","082", KC.samples)
KC.samples<-gsub("83-","083", KC.samples)
KC.samples<-gsub("91-","091", KC.samples)
KC.samples<-gsub("92-","092", KC.samples)
KC.samples<-gsub("93-","093", KC.samples)
#KC.sample.no<- substr(rownames(KCfs@phenoData@data), 14, 16)

##### 3.2 COMBINE THIS INFO AND WRITE TO CSV #####
#YI
YI.info<-data.frame(YI.names,
YI.samples,YI.sample.nos,YI.days,YI.sites,YI.plant,YI.stain)
colnames(YI.info)<-c("name","Sample ID","Sample
No.","Day","Site","Plant","Stain") #NB the 'name' column (matching
rownames in pData(Xfs) must be present in the X.info df for ggplot to use
this info
write.csv(YI.info, file="YI.basic_metadata.csv")
write.csv(YIfs@phenoData@data, file="YI.phenodata.csv")
#RB
RB.info<-data.frame(RB.names,
RB.samples,RB.sample.nos,RB.days,RB.sites,RB.plant,RB.stain)
colnames(RB.info)<-c("name","Sample ID","Sample
No.","Day","Site","Plant","Stain")
write.csv(RB.info, file="RB.basic_metadata.csv")
```

```
#KC
KC.info<-data.frame(KC.names,
KC.samples,KC.sample.nos,KC.days,KC.sites,KC.plant,KC.stain)
colnames(KC.info)<-c("name","Sample ID","Sample
No.","Day","Site","Plant","Stain")
write.csv(KC.info, file="KC.basic_metadata.csv")

##### 3.3 MATCH METADATA ROWNAMES WITH "phenoData@data" #####
row.names(YI.info)<-row.names(YIfs@phenoData@data)
row.names(RB.info)<-row.names(RBfs@phenoData@data)
row.names(KC.info)<-row.names(KCfs@phenoData@data)

##### 3.4 REPLACE "phenoData@data" WITH METADATA #####
YIfs@phenoData@data<-YI.info
RBfs@phenoData@data<-RB.info
KCfs@phenoData@data<-KC.info

##### 3.5 MATCH "phenoData@varmetadata" DIMS WITH METADATA #####
# YI DATASET
YI.VMDrows<-nrow(YIfs@phenoData@varMetadata)
YI.MDrows<-ncol(YI.info)
YI.rowstoadd<-YI.MDrows-YI.VMDrows
YIfs@phenoData@varMetadata[nrow(YIfs@phenoData@varMetadata)+YI.rowstoadd,
] <- NA
# RB DATASET
RB.VMDrows<-nrow(RBfs@phenoData@varMetadata)
RB.MDrows<-ncol(RB.info)
RB.rowstoadd<-RB.MDrows-RB.VMDrows
RBfs@phenoData@varMetadata[nrow(RBfs@phenoData@varMetadata)+RB.rowstoadd,
] <- NA
# KC DATASET
KC.VMDrows<-nrow(KCfs@phenoData@varMetadata)
KC.MDrows<-ncol(KC.info)
KC.rowstoadd<-KC.MDrows-KC.VMDrows
KCfs@phenoData@varMetadata[nrow(KCfs@phenoData@varMetadata)+KC.rowstoadd,
] <- NA

##### 3.6 RENAME ROWNAMES IN "fs@phenodata@varmetadata" TO MATCH COLNAMES
IN METADATA #####
row.names(YIfs@phenoData@varMetadata)<-colnames(YI.info)
row.names(RBfs@phenoData@varMetadata)<-colnames(RB.info)
row.names(KCfs@phenoData@varMetadata)<-colnames(KC.info)

##### 4 COMBINE FLOWSETS #####
allplantfs<-rbind2(YIfs,RBfs) # since rbind2 only accepts 2 objects per
command will need to run two rbind2's
allplantfs<-rbind2(allplantfs,KCfs)

##### 5 LGCL transformation of ... #####
##### 5.1 CREATE GLOBAL FRAME FOR LGCL ESTIMATION #####
# The following getGlobalFrame function was kindly provided by Radina
Droumeva
getGlobalFrame<- function(fs, length = 10, r = 5){
  if (is(fs,'flowFrame')){
    return (frame)
  }
  n <- length(fs)
  sample.n<-min(length,n)
```

```
  global.frame <- fsApply(fs[sample(n, sample.n)],
                          function(frame){
                            m <- nrow(frame)
                            frame <- frame[sample(m, min(m, m*r
                                                  /sample.n))]
                            return (frame)
                          })
  global.frame <- as(global.frame, 'flowFrame')
  return (global.frame)
}
#global.frame<-getGlobalFrame (YIfs)
global.frame<-getGlobalFrame (allplantfs)
##### 5.2 ESTIMATE LGCL PARAMETERS #####
lgcl <-estimateLogicle(global.frame, channels =c(fl1h, fl2h, fl3h, fl4h,
fl1a, fl2a, fl3a, fl4a, ssca, ssch, fsca, fsch), type="data")

##### 5.3 TRANSFORM FLOW SETS #####
#allplant.trans<-transform(YIfs,lgcl)
allplant.trans<-transform(allplantfs,lgcl)
###### 6 CONVERT FLOWSET TO NCDF OBJECT (allow >1 hr to run) #####
allplant_ncdf<-ncdfFlowSet(allplant.trans)

###### 7 OpenCyto ANALYSIS ####
##### 7.1 COMPUTATIONAL GATING USING A GATING TEMPLATE #####
gs<-GatingSet(allplant_ncdf)
gt<-gatingTemplate("C:/../gating_template.csv")
gt_gating(gt,gs)
#to obtain the index number of specific samples to plot
#names(keyword(gs))

##### 7.2 PLOTING OF SOME SAMPLES FOR CONTROL #####
plotGate(gs[[350]],xbin=256)
plotGate(gs[21:40],"singlets", default.y='FSC-H', xlim=c(4,8.5),
ylim=c(4,8.5),margin=FALSE,xbin=256)
plotGate(gs[946:956],"SYBR_Green_pos", default.y='FSC-
H',margin=FALSE,xbin=64)
plotGate(gs[155:175],"PI_pos", default.y='FSC-H',margin=FALSE,xbin=64)

# if plotGate is empty, please use ggcyto command, for example:
ggcyto(gs[946:956], aes(x = `FL1-H`, y = `SSC-H`) ,max_nrow_to_plot =
Inf) + geom_hex(bins = 128) +geom_gate("SYBR_Green_pos")+
theme(legend.position='right')+ geom_stats()

##### 7.3 EXTRACTION OF GATING DATA STATISTICS #####
freq_gs<-getPopStats(gs,statistic="freq", format='wide')
count_gs<-getPopStats(gs,statistic="count", format='wide')
#ploting of gating template
plot(gt)
#extract stats
stats = getPopStats(gs)
#calculate props
stats[,prop := Count/ParentCount]
#incorporate metadata
stats = merge(stats,pData(gs),by="name")
stats
write.csv(stats, 'allplant_flow_stats_july2020.csv')

##### 8 DATA MERGING #####
```

```
##### 8.1 IMPORT SCADA AND CHEMISTRY DATA #####
plant_data <- read.table("C:/../DCP metadata.csv",sep=",", header= TRUE,
na.strings="NA")##### 8.2 SUBSET OF SINGLETS DATA (TOTAL CELLS COUNT) AND
ADD OF PLANT METADATA ####
total_count<-stats[stats$Population=="/non_boundary/singlets",]
total_count <- merge(total_count,plant_data, by.x="Sample ID",
by.y="Sample_ID")
total_count$WCT_no_factor <- as.factor(total_count$WCT_no)
write.csv(total_count, 'all_count_data_stats_july2020.csv')

##### 8.3 SUBSET OF TOTAL COUNT, PI+ve AND SG+ve COUNT AND RATIO
pi_pos<-stats[stats$Population=="/non_boundary/singlets/PI_pos" &
stats$Stain=="PI",]
colnames(pi_pos)[4] <- "PI_ve_count"
colnames(pi_pos)[5] <- "PI_parent_count"
colnames(pi_pos)[6] <- "PI_ve_ratio"
sg_pos<-stats[stats$Population=="/non_boundary/singlets/SYBR_Green_pos" &
stats$Stain=="SG",]
colnames(sg_pos)[4] <- "SG_ve_count"
colnames(sg_pos)[5] <- "SG_parent_count"
colnames(sg_pos)[6] <- "SG_ve_ratio"
living_count<- cbind (pi_pos, SG_ve_count=sg_pos$SG_ve_count [match
(pi_pos$`Sample ID`,sg_pos$`Sample ID`)])
living_count<- cbind (living_count,
SG_parent_count=sg_pos$SG_parent_count [match (pi_pos$`Sample
ID`,sg_pos$`Sample ID`)])
living_count<- cbind (living_count, SG_ve_ratio=sg_pos$SG_ve_ratio [match
(pi_pos$`Sample ID`,sg_pos$`Sample ID`)])

##### 8.4 CALCULATE LIVING CELL STATS (ICCs) #####
# POPmcc = pi+ve ratio / SG+ve ratio
# POPicc = 1 - POPmcc
# ICC = POPicc * SG+ve count
living_count$dead_cell_ratio <-
(living_count$PI_ve_ratio/living_count$SG_ve_ratio)
living_count$living_cell_ratio <- (1 - living_count$dead_cell_ratio)
living_count$living_cell_count <- (living_count$living_cell_ratio *
living_count$SG_ve_count)
living_count$living_cell_ml <- (living_count$living_cell_count * 40)
living_count <- merge(living_count,plant_data, by.x="Sample ID",
by.y="Sample_ID")

##### 8.5 REMOVE REDUNDANT COLUMNS AND TIDY UP DF #####
living_count<-living_count[,c(-17, -18)]
lc_cols<-colnames(living_count)
lc_cols[4:5]<-c("Site", "Plant")
colnames(living_count)<-lc_cols
living_count$WCT_no_factor <- as.factor(living_count$WCT_no)
living_count$plant_performance_invert <-
1/(living_count$Plant_performance_.kW.Tr.)
living_count$chiller_performance_invert <-
1/(living_count$Chiller_performance)

##### 8.6 ADD MEAN TOTAL COUNT #####
mean_total_count <- aggregate (x=total_count$Count,
by=list(total_count$`Sample ID`), FUN=mean)
colnames(mean_total_count)[1] <- "Sample ID"
colnames(mean_total_count)[2] <- "Mean_count"
```

```
living_count<- cbind (living_count,
mean_count=mean_total_count$Mean_count [match (living_count$`Sample
ID`,mean_total_count$`Sample ID`)])
write.csv(living_count, 'living_data_stats_july2020_new.csv')

###### 8.7 SUBSET OF DATA FOR THE DIFFERENT SITE AND PLANT FOR
STATISTICAL ANALYSIS #####
A_site_count <- living_count[living_count$Site.y=="A",]
BC_site_count <-
living_count[living_count$Site.y=="C"|living_count$Site.y=="B",]
B_site_count <- living_count[living_count$Site.y=="B",]
C_site_count <- living_count[living_count$Site.y=="C",]
KC_C_living_count<-C_site_count[C_site_count$Plant.x=="KC",]
RB_C_living_count<-C_site_count[C_site_count$Plant.x=="RB",]
YI_C_living_count<-C_site_count[C_site_count$Plant.x=="YI",]

#SessionInfo
# R version 3.6.1 (2019-07-05)
# Platform: x86_64-w64-mingw32/x64 (64-bit)
# Running under: Windows 10 x64 (build 18362)
#
# Matrix products: default
#
# locale:
#   [1] LC_COLLATE=English_United Kingdom.1252  LC_CTYPE=English_United
Kingdom.1252    LC_MONETARY=English_United Kingdom.1252
# [4] LC_NUMERIC=C                            LC_TIME=English_United
Kingdom.1252
#
# attached base packages:
#   [1] grid      parallel  stats     graphics  grDevices utils
datasets  methods   base
#
# other attached packages:
#   [1] Rgraphviz_2.28.0          graph_1.62.0
BiocGenerics_0.30.0       openCyto_1.24.0           ggcyto_1.15.0
# [6] ggplot2_3.2.1             flowViz_1.48.0            lattice_0.20-38
flowStats_3.42.0          ncdfFlow_2.32.0
# [11] BH_1.69.0-1              RcppArmadillo_0.9.800.1.0
flowWorkspace_3.35.0      cluster_2.1.0             flowCore_1.52.0
#
# loaded via a namespace (and not attached):
#   [1] mclust_5.4.5        Rcpp_1.0.3          mvtnorm_1.0-11
corpcor_1.6.9       gtools_3.8.1        assertthat_0.2.1    digest_0.6.23
# [8] R6_2.4.1            plyr_1.8.4          stats4_3.6.1
pcaPP_1.9-73        ellipse_0.4.1       pillar_1.4.2
zlibbioc_1.30.0
# [15] rlang_0.4.1         lazyeval_0.2.2      rstudioapi_0.10
data.table_1.12.6   hexbin_1.28.0       R.oo_1.22.0         R.utils_2.9.0
# [22] Matrix_1.2-17       splines_3.6.1       stringr_1.4.0
munsell_0.5.0       compiler_3.6.1      pkgconfig_2.0.3     mnormt_1.5-5
# [29] IDPmisc_1.1.19      tidyselect_0.2.5    tibble_2.1.3
gridExtra_2.3       matrixStats_0.55.0  rrcov_1.4-7         crayon_1.3.4
# [36] dplyr_0.8.3         withr_2.1.2         R.methodsS3_1.7.1
MASS_7.3-51.4       RBGL_1.60.0         gtable_0.3.0        magrittr_1.5
# [43] scales_1.0.0        RcppParallel_4.4.4  KernSmooth_2.23-15
stringi_1.4.3       latticeExtra_0.6-28 robustbase_0.93-5
RColorBrewer_1.1-2
```

```
# [50] tools_3.6.1          Biobase_2.44.0      glue_1.3.1
DEoptimR_1.0-8      purrr_0.3.3         ks_1.11.5           clue_0.3-57
# [57] colorspace_1.4-1    flowClust_3.22.0    fda_2.4.8
```
